# Targets Fishing and Identification of Calenduloside E as Hsp90AB1: Design, Synthesis, and Evaluation of Clickable Activity-Based Probe

**DOI:** 10.3389/fphar.2018.00532

**Published:** 2018-05-23

**Authors:** Shan Wang, Yu Tian, Jing-Yi Zhang, Hui-Bo Xu, Ping Zhou, Min Wang, Sen-Bao Lu, Yun Luo, Min Wang, Gui-Bo Sun, Xu-Dong Xu, Xiao-Bo Sun

**Affiliations:** ^1^Beijing Key Laboratory of Innovative Drug Discovery of Traditional Chinese Medicine (Natural Medicine) and Translational Medicine, Institute of Medicinal Plant Development, Chinese Academy of Medical Sciences & Peking Union Medical College, Beijing, China; ^2^Academy of Chinese Medical Sciences of Jilin Province, Changchun, China; ^3^Department of Bioengineering, Santa Clara University, Santa Clara, CA, United States; ^4^Life and Environmental Science Research Center, Harbin University of Commerce, Harbin, China

**Keywords:** Calenduloside E, clickable activity based protein profiling, computational chemistry, HUVECs, Hsp90AB1

## Abstract

Calenduloside E (CE), a natural triterpenoid compound isolated from *Aralia elata*, can protect against ox-LDL-induced human umbilical vein endothelial cell (HUVEC) injury in our previous reports. However, the exact targets and mechanisms of CE remain elusive. For the sake of resolving this question, we designed and synthesized a clickable activity-based probe (CE-P), which could be utilized to fish the functional targets in HUVECs using a gel-based strategy. Based on the previous studies of the structure-activity relationship (SAR), we introduced an alkyne moiety at the C-28 carboxylic group of CE, which kept the protective and anti-apoptosis activity. Via proteomic approach, one of the potential proteins bound to CE-P was identified as Hsp90AB1, and further verification was performed by pure recombinant Hsp90AB1 and competitive assay. These results demonstrated that CE could bind to Hsp90AB1. We also found that CE could reverse the Hsp90AB1 decrease after ox-LDL treatment. To make our results more convincing, we performed SPR analysis and the affinity kinetic assay showed that CE/CE-P could bind to Hsp90AB1 in a dose-dependent manner. Taken together, our research showed CE could probably bind to Hsp90AB1 to protect the cell injury, which might provide the basis for the further exploration of its cardiovascular protective mechanisms. For the sake of resolving this question, we designed and synthesized a clickable activity-based probe (CE-P), which could be utilized to fish the functional targets in HUVECs using a gel-based strategy.

## Introduction

Natural products represent an enormous source of pharmacologically useful compounds and are often used as the starting point in modern drug discovery. However, many biologically interesting natural products are not being pursued as potential drug candidates, partly due to the lack of well-defined mechanisms of action. The identification of drug targets is very important in the process of drug discovery, which allows researchers to clarify the mechanisms of drug action (Krysiak and Breinbauer, 2012; Yue et al., 2012). Activity-based protein profiling (ABPP) is a chemical proteomic method that uses active site-directed chemical probes to selectively target subsets of proteins in the proteome based on shared mechanistic and/or structural features (Barglow and Cravatt, 2007; Cravatt et al., 2008; Pichler et al., 2016). This technique has been widely used in enzyme proteomes with quantitative proteomics development; this technique has been used to identify unknown target compounds (Chen et al., 2017). The basic chemical structure of the molecular probe consists of three parts: a reactive group, a binding group, and a reporter tag. The ABPP probe targets a large number of proteins via the reactive group, providing researchers with a global view of the proteome profile. Then, target proteins are identified by quantitative proteomics analysis (Hunerdosse and Nomura, 2014; Wright and Sieber, 2016). However, most tags are relatively bulky compared with the small molecule probe, which influences cell permeability and may prevent the reactive group from entering the active site. With the development of click chemistry, CC-ABPP strategies using a biorthogonal reaction with a label-free probe have been increasingly applied to circumvent this issue (Speers and Cravatt, 2009; Li et al., 2012). The reporter group is substituted with a small, latent chemical handle (alkyne or azide), which does not impede cell permeability, and can be simultaneously diversified with a variety of reporter groups without the need to develop new synthetic routes (Martell and Weerapana, 2014). The CC-ABP probe has advanced the ABPP field by expanding the enzyme classes targeted by ABPs, enabling cellular and *in vivo* studies and providing technological platforms to quantitatively monitor protein activities in complex biological systems. Currently, the CC-ABPP technology has become an effective method for the discovery of functional targets of small molecules (Lapinsky and Johnson, 2015).

**Figure 1:**
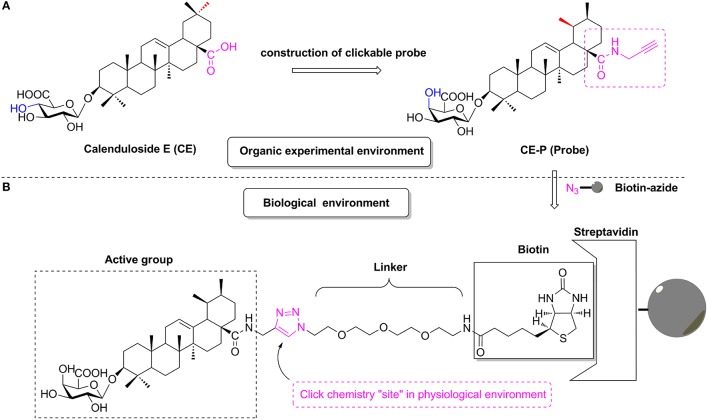
The design of CC-ABPP **CE-P** from lead compound Calenduloside E (CE). **(A)** Construction of clickable probe CE-P in organic experimental environment. **(B)** The probe CE-P coupled with biotin moiety in physiological environment.

*Aralia elata* (Miq) Seem (AS), which is used extensively in traditional Chinese medicine (TCM), has been used as a tonic herb due to its anti-arrhythmic, anti-arthritic, anti-hypertensive and anti-diabetic effects (Baranov, 1982). Moreover, as a main component of *A. elata* Xinmaitong capsules (Clinical Trial Approval Number 2003L01111 by the China Food and Drug Administration), AS was developed for the treatment of coronary heart disease and has successfully completed Phase III clinical trials in China. According to our previous studies, AS exhibited anti-myocardial ischemia and anti-hypoxia activities (Wang et al., 2014, 2015, 2017). The total saponins from AS are considered the main pharmacologically active ingredients of AS. Various oleanane-type triterpene saponins were extracted from AS and identified. Calenduloside E (CE, Figure 1) is one of the natural triterpene saponins extracted from AS. Calenduloside E was previously shown to protect endothelial cells from injury and reduce apoptotic endotheliocytes and it could protect against H_2_O_2_-induced H9c2 cardiomyocytes apoptosis (Tian et al., 2017a,b). Using the ABPP probe, we identified 587 proteins as the most likely targets of CE. In our previous paper, our ABPP probe was the basic probe, but the biotin tag on the probe may have interfered with the binding of CE to the targets. In our present research, for the first time, we designed and synthesized a clickable probe CE-P, which could be introduced the biotin tag via click chemistry to avoid the interference of bulk molecule. Utilizing this CC-ABPP strategy, we identified and confirmed potential targets of CE.

## Materials and methods

### Materials

ox-LDL was obtained from Union-Biotechnology. Annexin-V/Propidium iodide (FITC/PI) staining kit (V13241) was Molecular Probes™. MTT [3-(4, 5-dimethylthiazol-2-yl)-2, 5-diphenyltetrazoliumbromide, 0973] was the products of Amresco. JC-1 (C2005) was purchased from Beyotime biotechnology. Caspase-3 fluorometric assay kit (K105-200) was acquired from BioVision. VascuLife® VEGF Endothelial Cell Culture Medium (LL-0003) was the products of Lifeline cell technology. TBTA (Tris[(1-benzyl-1H-1, 2, 3-triazol-4-yl)methyl]-amineT2993), TCEP (Tris(2-carboxyethyl)phosphine, T1656) were purchased from Tokyo Chemical Industry. Biotin-azide was provided from the Institute of Medicinal Plant Development (Beijing, China) (Tian et al., 2017b). HOBt (N-Hydroxybenzotriazole), EDCI (1-Ethyl-(3-dimethylaminopropyl) carbodiimide hydrochloride), TEMPO (2, 2, 6, 6-Tetramethylpiperidine1-oxyl) were purchased from Energy Chemical Industry. Pierce™ Streptavidin Agarose (20347), Pierce™ Silver Stain for Mass Spectrometry (24600) was from Thermo Fisher Scientific. The primary antibody against Hsp90AB1, Bcl2, Cytochrome C was obtained from Santa Cruz Biotechnology (Santa Cruz, CA, USA). Lox1 primary antibody was from Abcam (Cambridge, UK).Recombinant human Hsp90AB1 protein was from Abcam (Cambridge, UK).

### Chemistry

Glycosyl donor **compound i** was prepared from galactose, and the reaction conditions were reported previously by Schmidt (Sun et al., 2014).

#### Synthesis of compound I

To a solution of ursolic acid (10.0 g, 21.8 mmol) in dry DCM (300 mL), TBAB (0.8 g, 2.5 mmol) and K_2_CO_3_ (7.4 g, 53.6 mmol) in water (50 mL) were added, and benzyl bromide (3.2 mL, 26.8 mmol) was dropped at 0°C. Then the reaction mixture was stirred at room temperature for 18 h. Reaction was monitored by TLC. The crude mixture was separated and the water layer was extracted with DCM (3 × 100 mL). The combined organic layer was washed with 0.1 mol/L HCl aqueous solution, NaHCO_3_ saturated aqueous solution and NaCl saturated aqueous solution in sequence, and then dried over Na_2_SO_4_ and purified through column chromatography (eluent: PE-EtOAc, 8:1) to offer pure white solid **compound I** (11.1 mg, 93% yield). ^1^H-NMR (600 MHz, pyridine-*d*_5_) δ: 7.36–7.29 (m, 5H, OPh-H), 5.23 (t, *J* = 3.3 Hz, 1H, H-12), 5.10 (d, *J* = 12.5 Hz, 1H, C*H*_2_OPh), 4.98 (d, *J* = 12.5 Hz, 1H, C*H*_2_OPh), 3.23–3.19 (m, 1H, H-3), 2.26 (d, *J* = 11.1 Hz, 1H, H-18), 1.07 (s, 3H, CH_3_), 0.98 (s, 3H, CH_3_), 0.93 (d, *J* = 6.3 Hz, 3H, CH_3_), 0.89 (s, 3H, CH_3_), 0.85 (d, *J* = 6.5 Hz, 3H, CH_3_), 0.77 (s, 3H, CH_3_), 0.64 (s, 3H, CH_3_); ^13^C-NMR (150 MHz, pyridine-*d*_5_) δ: 177.5, 138.2, 136.5, 128.5, 128.3, 128.1, 125.8, 79.2, 77.4, 77.2, 76.9, 66.1, 55.3, 53.0, 48.2, 47.7, 42.2, 39.6, 39.2, 39.0, 38.9, 38.7, 37.1, 36.8, 33.1, 30.8, 28.3, 28.1, 27.3, 24.4, 23.7, 23.4, 21.3, 18.4, 17.1, 15.8, 15.6.

#### Synthesis of compound II

To a solution of **compound I** (3.3 g, 6.0 mmol) in dry DCM (50 mL), glycosyl donor **compound i** (5.8 g, 7.9 mmol) and 4Å molecular sieve 0.5 g were added and stirred at room temperature for 1 h under N_2_ air. Then lewis acid TMSOTf (60 μg, 0.3 mmol) was dropped and reacted for 2–4 h. When complete, triethylamine 1.0 mL was added to quench the reaction. Then the suspension was filtered out and the filtrate was evaporated and the crude product was subjected to column chromatography (eluent: PE-EtOAc, 10:1) to gain pure **compound II** (4.7 g, 70% yield) as white solid. ^1^H-NMR (600 MHz, pyridine-*d*_5_) δ: 8.25–8.22 (m, 4H, OBz-H), 8.16–8.15 (m, 2H, OBz-H), 8.01–8.00 (m, 2H, OBz-H), 7.56–7.52 (m, 3H, OBz-H), 7.49–7.41 (m, 6H, OBz-H), 7.38–7.34 (m, 3H, OBz-H), 7.29–7.27 (m, 3H, OBz-H), 7.10–7.08 (m, 2H, OBz-H), 6.55–6.54 (m, 1H, Gal-H), 6.48–6.45 (m, 1H, Gal-H), 6.40–6.38 (m, 1H, Gal-H), 5.43 (d, *J* = 7.9 Hz, 1H, Glc-H-1′), 5.41 (t, *J* = 3.3 Hz, 1H, H-12), 5.34 (d, *J* = 12.5 Hz, 1H, OBn-H), 5.22 (d, *J* = 12.4 Hz, 1H, OBn-H), 5.16–5.13 (m, 1H, Gal-H), 4.96–4.94 (m, 1H, Gal-H), 4.80–4.77 (m, 1H, Gal-H), 3.39 (dd, *J* = 11.9 Hz, 4.4 Hz, 1H, H-3), 2.47 (d, *J* = 11.3 Hz, 1H, H-18), 1.15 (s, 3H, CH_3_), 0.97 (d, *J* = 6.5 Hz, 3H, CH_3_), 0.93 (s, 6H, 2×CH_3_), 0.79 (s, 3H, CH_3_), 0.77 (s, 3H, CH_3_), 0.73 (s, 3H, CH_3_); ^13^C-NMR (150 MHz, pyridine-*d*_5_) δ: 176.8, 166.1, 166.0,165.8, 165.7, 138.5, 137.1, 133.8, 133.6, 133.5, 133.4, 130.3, 130.1, 130.0, 129.9, 129.8, 129.5, 129.0, 128.8, 128.7, 128.5, 128.3, 125.9, 103.8, 90.2, 72.6, 71.7, 71.1, 69.3, 66.1, 62.7, 55.5, 53.3, 48.2, 47.7, 42.2, 39.7, 39.2, 39.0, 38.9, 38.5, 36.9, 36.6, 33.2, 30.7, 28.2, 27.9, 26.4, 24.5, 23.7, 23.4, 21.2, 18.2, 17.2, 17.1, 16.6, 15.3.

#### Synthesis of compound III

A mixture of **compound II** (3.0 g, 2.6 mmol) and 10% Pd/C (1.5 mg) was hydrogenated at 1 atm for 4–6 h in refluxing EtOAc (30 mL). The mixture was filtered and concentrated, the residue was purified by silica gel column chromatography (eluent: PE-EtOAc, 3:1) to get pure **compound III** (2.4 g, 91% yield) as white solid. ^1^H-NMR (600 MHz, pyridine-*d*_5_) δ: 8.24–8.21 (m, 4H, OBz-H), 8.14–8.13 (m, 2H, OBz-H), 8.00–7.98 (m, 2H, OBz-H), 7.56–7.53 (m, 1H, OBz-H), 7.48–7.45 (m, 2H, OBz-H), 7.43–7.41 (m, 2H, OBz-H), 7.38–7.35 (m, 2H, OBz-H), 7.28–7.26 (m, 3H, OBz-H), 7.10–7.07 (m, 2H, OBz-H), 6.54–6.53 (m, 1H, Gal-H), 6.46–6.43 (m, 1H, Gal-H), 6.39–6.36 (m, 1H, Gal-H), 5.50 (t, *J* = 3.3 Hz, 1H, H-12), 5.42 (d, *J* = 7.9 Hz, 1H, Glc-H-1′), 5.15–5.12 (m, 1H, Gal-H), 4.96–4.93 (m, 1H, Gal-H), 4.79–4.76 (m, 1H, Gal-H), 3.38 (dd, *J* = 11.7 Hz, 4.3 Hz, 1H, H-3), 2.65 (d, *J* = 11.3 Hz, 1H, H-18), 1.22 (s, 3H, CH_3_), 1.05 (d, *J* = 6.4 Hz, 3H, CH_3_), 0.98–0.97 (m, 6H, 2×CH_3_), 0.92 (s, 3H, CH_3_), 0.76 (s, 3H, CH_3_), 0.72 (s, 3H, CH_3_); ^13^C-NMR (150 MHz, pyridine-*d*_5_) δ: 179.8, 166.1, 166.0, 165.8, 165.7, 139.1, 133.8, 133.6, 133.6, 133.4, 130.3, 130.0, 130.0, 130.0, 129.8, 129.4, 129.4, 129.0, 128.8, 128.8, 128.6, 125.4, 103.7, 90.2, 72.5, 71.7, 71.0, 69.3, 62.7, 55.7, 53.4, 47.9, 47.8, 43.3, 42.3, 39.7, 39.4, 39.3, 38.9, 38.5, 37.3, 36.6, 33.3, 30.9, 28.5, 27.8, 26.4, 24.8, 23.8, 23.4, 21.3, 18.2, 17.4, 17.2, 16.6, 15.3.

#### Synthesis of compound IV

To a solution of compound **III** (1.0 g, 0.98 mmol) in dry DCM (15 mL), HOBt (0.2 g, 1.46 mmol) and EDCI (0.28 g, 1.46 mmol) were added and stirred at room temperature for 1 h. To this mixture, propargylamine (0.22 g, 3.92 mmol) was added respectively at 0°C and the reaction mixture was stirred until its completion for 8 h. The solvent was washed with 0.1 mol/L HCl aqueous solution, NaHCO_3_ saturated aqueous solution and NaCl saturated aqueous solution in sequence, and then dried over Na_2_SO_4_. The suspension was filtered and the filtrate was concentrated and purified through column chromatography (eluent: DCM-CH_3_OH, 100:1) to offer pure white solid compound **IV** as white solid, 79% yield. ^1^H-NMR (600 MHz, pyridine-*d*_5_) δ: 8.24–8.22 (m, 4H, OBz-H), 8.14–8.13 (m, 2H, OBz-H), 7.99–7.98 (m, 2H, OBz-H), 7.94 (t, 1H, CONH), 7.53–7.52 (m, 1H, OBz-H), 7.47–7.46 (m, 2H, OBz-H), 7.43–7.40 (m, 2H, OBz-H), 7.37–7.35 (m, 2H, OBz-H), 7.28–7.26 (m, 3H, OBz-H), 7.09–7.07 (m, 2H, OBz-H), 6.54 (m, 1H, Gal-H), 6.46–6.37 (m, 2H, Gal-H), 5.47–5.42 (m, 2H, H-12, Glc-H-1′), 5.15–5.12 (m, 1H, Gal-H), 4.95 (m, 1H, Gal-H), 4.79–4.77 (m, 1H, Gal-H), 4.33 (m, 2H, CONHCH_2_), 3.39 (m, 1H, H-3), 3.13 (m, 1H, CCH), 2.47 (d, *J* = 10.3 Hz, 1H, H-18), 1.19 (s, 3H, CH_3_), 1.00 (d, *J* = 5.2 Hz, 3H, CH_3_), 0.97–0.92 (m, 9H, 3×CH_3_), 0.78–0.77 (m, 6H, 2×CH_3_); ^13^C-NMR (150 MHz, pyridine-*d*_5_) δ: 177.1, 166.1, 166.0, 165.8, 165.7, 139.2, 133.8, 133.6, 133.6, 133.4, 130.3, 130.0, 130.0, 130.0, 129.8, 129.4, 129.4, 129.0, 128.8, 128.8, 128.6, 125.8, 103.7, 90.2, 81.9, 72.5, 71.9, 71.7, 71.0, 69.3, 62.7, 55.5, 53.1, 47.8, 47.7, 43.3, 42.3, 39.8, 39.7, 39.2, 38.9, 38.5, 37.7, 36.6, 33.1, 31.0, 29.9, 29.1, 28.1, 27.8, 26.4, 24.7, 23.7, 23.5, 21.3, 18.2, 17.5, 17.4, 16.6, 15.3.

#### Synthesis of compound V

To a solution of compound **IV** in MeOH/DCM (8 mL, 3:1) was added 1 mol/L NaOMe/NaOH solvent (1.6 mL). The reaction mixture was stirred for 2 h until its completion, after that Amberlite IR-120 was added to acidate PH 7. The suspension was filtered out and the filtrate was evaporated and purified through column chromatography (eluent: DCM-CH_3_OH, 10:1) to offer pure white solid compound **V** as white solid, 93% yield. ^1^H-NMR (600 MHz, pyridine-*d*_5_) δ: 7.84 (t, *J* = 5.4 Hz, 1H, N-H), 5.45 (t, *J* = 3.3 Hz, 1H, H-12), 4.89 (d, *J* = 7.7 Hz, 1H, H-1′), 4.60–4.59 (m, 1H, Gal-H), 4.52–4.46 (m, 3H, Gal-H), 4.40–4.28 (m, 2H, H-31), 4.19 (dd, *J* = 3.4 Hz, 9.4 Hz, 1H, Gal-H), 4.13 (t, *J* = 6.2 Hz, 1H, Gal-H), 3.43 (dd, *J* = 11.8 Hz, 4.5 Hz, 1H, H-3), 3.10 (t, *J* = 2.4 Hz, 1H, H-33), 2.44 (d, J = 10.8 Hz, 1H, H-18), 1.34 (s, 3H, CH_3_), 1.24 (s, 3H, CH_3_), 1.02 (s, 3H, CH_3_), 1.00 (s, 3H, CH_3_), 0.97 (d, *J* = 6.5 Hz, 3H, CH_3_), 0.94 (s, 3H, CH_3_), 0.90 (s, 3H, CH_3_); ^13^C-NMR (150 MHz, pyridine-*d*_5_) δ: 177.2, 139.4, 126.0, 107.5, 88.8, 81.9, 76.8, 75.4, 73.1, 71.9, 70.3, 62.5, 55.9, 53.3, 47.9, 47.8, 42.5, 40.0, 39.8, 39.5, 39.3, 38.9, 37.8, 36.8, 33.3, 31.1, 29.2, 28.3, 26.7, 24.8, 23.8, 23.6, 21.3, 18.4, 17.6, 17.4, 17.0, 15.6; HRMS (ESI): Calcd for [M + H]^+^ C_39_H_62_NO_7_: 656.4526, found 656.4516.

#### Synthesis of compound VI (CE-P)

To a solution of compound **V** (200.0 mg, 305.14 mmol) in DCM (1 mL), KBr (7.26 mg, 61.03 mmol), TEMPO (0.95 mg, 6.1 mmol) and TBAB (19.67 mg, 61.03 mmol) were added at room temperature. To a solution of this mixture was added Na_2_CO_3_/NaHCO_3_ (3 mL, PH 9.5). To this mixture, Ca(ClO)_2_ (87.26 mg, 610.28 mmol) was added at 0°C and the reaction mixture was stirred violently until its completion. The Na_2_SO_3_ 20 mg was added to quench the reaction, and then 6N HCl was dropped to acidate PH 3. The crude mixture was extracted with DCM (3 × 15 mL) and the combined organic layer was dried over Na_2_SO_4_ and purified through column chromatography (eluent: CH_2_Cl_2_-CH_3_OH-H_2_O, 50:10:1) to offer pure compound **VI** as white solid. (63.4 mg, 31% yield). ^1^H-NMR (600 MHz, pyridine-*d*^5^) δ: 7.91 (t, *J* = 5.4 Hz, 1H, N-H), 5.48 (t, *J* = 3.4 Hz, 1H, H-12), 4.87 (d, *J* = 7.7 Hz, 1H, H-1′), 4.65–4.58 (m, 1H, Gal-H), 4.56–4.46 (m, 1H, Gal-H), 4.46–4.28 (m, 3H, H-31, Gal-H), 4.24–4.12 (m, 1H, Gal-H), 3.43 (m, 1H, H-3), 3.11 (s, 1H, H-33), 2.48 (m, 14H, H-18), 1.36 (s, 3H, CH_3_), 1.25 (s, 3H, CH_3_), 1.02 (s, 3H, CH_3_), 0.99 (s, 3H, CH_3_), 0.97 (s, 6H, 2×CH_3_), 0.89 (s, 3H, CH_3_); ^13^C-NMR (150 MHz, pyridine-*d*^5^) δ: 177.1, 175.9, 139.3, 125.8, 107.0, 88.3, 81.9, 76.1, 75.9, 72.7, 72.5, 71.9, 55.8, 53.2, 47.8, 47.7, 42.4, 39.9, 39.9, 39.7, 39.4, 39.2, 37.7, 36.8, 33.4, 31.1, 29.9, 29.2, 28.9, 28.2, 24.7, 23.8, 23.6, 21.3, 18.4, 17.5, 17.5, 16.9, 15.6; HRMS calcd mass for C_39_H_59_NNaO_8_ [M+Na]^+^ 692.4138, found 692.4145. The spectrograms of the compounds I–VI were shown in Electronic Supplementary Material (ESI).

### Biological studies

#### Cell preparation and culture

HUVECs were isolated from fresh human umbilical veins using 0.1% collagenase I, as previously described (Qin et al., 2015). After dissociation, the cells were collected and cultured in VascuLife® VEGF Endothelial Cell Culture Medium (Lifeline Cell Technology, MD, USA) supplemented with 100 U/mL penicillin and 100 μg/mL streptomycin. All cell cultures were maintained in a humidified 37°C incubator with 5% CO2, and the media were refreshed every 3 days. Cells at passages 3–7 were used in subsequent experiments. Neonatal umbilical cords were donated by the Maternal and Child Care Service Center in Beijing, China.

#### Cell viability assay

Cell viability was determined using the MTT (3-(4, 5-dimethylthiazol-2-yl)-2, 5-diphenyl tetrazolium, Amresco, 0973) assay as previously described (Tian et al., 2017b). Briefly, HUVECs were plated on 96-well plates at a density of 8 × 10^4^ cells/well and then grown at 37°C for 24 h. The treatment group cells were pretreated with CE-P/CE for 8 h, followed by treatment with ox-LDL (80 μg/mL, 24 h), the control group was pretreated with vehicle for 8 h then exposed without ox-LDL. Twenty microliters of MTT (5 mg/mL) were added to each well and incubated for 4 h. The medium was removed and the formazan crystals were dissolved with dimethyl sulfoxide (DMSO). The absorbance was measured at 570 nm on a microplate reader (TECAN Infinite M1000, Austria).

#### Assessments of cell apoptosis

HUVECs were incubated with ox-LDL (70 μg/mL, 24 h) and pretreated with BCEA for 8 h prior to the apoptosis assay. Double fluorescence staining was performed using an Annexin V-FITC/PI apoptosis staining kit (Molecular Probes™, V13241) according to the manufacturer's instructions to detect early apoptotic and necrotic cells. Cellular fluorescence was measured using flow cytometry with a FACS Calibur Flow Cytometer (BD Biosciences, USA).

#### Determination of ΔΨm

We used JC-1 (5, 5′, 6, 6′-tetrachloro-1, 1′, 3, 3′–tetraethyl benzimidazolyl carbocyanine iodide, Beyotime Biotechnology, (C2005) to analyze ΔΨm. HUVECs were cultured on coverslips, the ox-LDL was removed, and the cells were washed twice with warm PBS and incubated with JC-1 (2 μM final concentration) for 30 min in the dark. The cells were finally washed twice with PBS, and images were captured using an EVOS® FL fluorescence microscope (Thermo Fisher Scientific, USA).

#### Analysis of Caspase-3 activation

Caspase-3 activity was measured using a Fluorometric Assay Kit (BioVision, USA) according to the manufacturer's instructions. The samples were measured in a Fluoroskan Ascent FL fluorometer (Thermo Fisher Scientific, USA) using a 400 nm excitation wavelength and a 505 nm emission wavelength. The results are expressed as fold changes compared to the control.

#### Biotin–neutravidin pull-down assay

HUVECs were cultured in a T75 culture flask. HUVECs at 100% confluence were lysed in PBS buffer, and the protein concentration was adjusted to 2 mg/mL. For each experimental and control sample, 2 × 0.5 mL aliquots of the 2 mg/mL cell homogenate were transferred into microcentrifuge tubes. The experimental and control samples were incubated with 5 μL of 10 mg/mL CE-P or 5 μL of DMSO at room temperature for 1 h. Then, the proteomes were labeled with biotin-azide (100 μM), TCEP, 1 mM), TBTA, 100 μM), and CuSO4·5H2O (1 mM) for 1 h. Seven hundred fifty microliters of cold MeOH were added and sonicated for 3–4 sec using a probe sonicator (~30% power level) at 4°C to re-suspend the protein. The samples were then centrifuged for 4 min at 6,500 × g at 4°C and the supernatant was removed. The pellets were dissolved in PBS containing 1.2% SDS via sonication and then diluted with PBS containing 0.2% SDS. The samples were incubated with streptavidin beads for 2 h at room temperature and washed with PBS several times. Samples were denatured by heating in 2 × SDS-loading buffer and analyzed by SDS-PAGE. The resulting bands were visualized with Coomassie blue staining (Lee et al., 2014). Next, trypsin digestion was performed on selected visible protein bands.

#### Western blot

Cell extracts were lysed in RIPA lysis buffer (Beyotime, Shanghai, China) containing a 1% protease inhibitor cocktail (Roche, Basel, Switzerland) (Sun et al., 2012). The protein content was measured with a BCA Protein Assay Kit (CWBiotech, Beijing, China). Approximately 30–50 μg of protein were resolved using 10 or 12% SDS-PAGE and then transferred to polyvinylidene difluoride membranes. The membranes were incubated with 1:500-diluted primary antibodies overnight at 4°C, followed by horseradish peroxidase-conjugated secondary antibodies at room temperature. Then, the proteins were developed with an enhanced chemiluminescence detection system and imaged using a Bio-Rad imaging system (Bio-Rad, Hercules, CA, USA).

#### CE-P binds to recombinant Hsp90AB1

CE-P was incubated with the recombinant Hsp90AB1 protein at room temperature for 1 h. The protein was pulled down as the same as the previous described methods (**Biotin–neutravidin pull-down assay**), then was detected by silver staining (Thermo Fisher Scientific, USA).

#### Targets predicted by discovery Studio 2016

The molecular targets of CE-P were predicted using Discovery Studio 2016 (BIOVIA Software Inc., San Diego, CA, USA), a software suite for performing computational analysis of data relevant to Life Sciences research. To determine the probable target of CE-P, we employed the Ligand Profiler protocol which maps a set of pharmacophores, including Pharma DB by default. The ligand CE-P was prepared by the Specifying Ligands parameter protocol. After inputting all parameters, the job was run and the results were monitored from the Jobs Explorer.

#### Molecular docking

To explore the potential interacting mode of CE/CE-P with the Hsp90AB1 protein (PDB code: 3NMQ), a molecular modeling study was performed using the docking program named Induced-Fit, a refinement method in another software MOE. To eliminate any bond length and bond angle biases, the ligand (CE/CE-P) was subjected to an “energy minimize” prior to docking. The binding affinities (*S*-values) in MOE were used to evaluate the interactions between Hsp90AB1 and CE/CE-P. The scores (binding affinities) were obtained based on the virtual calculation of various interactions of the ligands with the targeted receptor.

#### Surface-plasmon resonance (SPR)

The molecule/protein interaction detection and kinetic constant measurement were studied using the Biacore System. CM5 Sensor Chip was activated using sulpho-NHS/EDC chemistry in a buffer consisting of 2.7 mM KCl 137 mM NaCl, 0.05% (v/v) surfactant P20, pH 7.4. The chip was subsequently immobilized with the recombinant human Hsp90AB1 protein at a concentration of 37 μg/ml in sodium acetate, pH 4.5 and then blocked with 1 M ethanolamine, pH 8.0. Compounds were dissolved to 10 mM in 100% DMSO and then 50-fold into running buffer without DMSO then diluted two-fold by running buffer into 12.5, 6.25, 3.125, 1.56, 0.78, and 0 μM before injection. The optical interference pattern was recorded as a change in optical path difference in units of nm. Data were analyzed with Biacore T200 Evaluation Software.

### Statistical analysis

Data are presented as the means ± standard deviation (*SD*) of three independent experiments. The groups were compared using one-way ANOVA followed by Tukey's multiple comparison tests using the statistics module of Graph Pad Prism 5.0. A value of *P* < 0.05 was considered statistically significant.

## Results

### Design and synthesis of the CC activity-based protein profiling probe CE-P based on CE

According to previous studies, the biotinylated probe BCEA, which maintains the active moiety of the parental compound CE, exhibits similar protective effects against ox-LDL-induced human umbilical vein endothelial cell (HUVEC) damage and identified 128 proteins related to cell survival signaling pathways as the targets (Tian et al., 2017b). Based on studies of the structure-activity relationship (SAR), amide derivatives of CE containing ursane and galactoside scaffolds maintained similar activity to the parental compound CE (Tian et al., 2017a,b). In the current study, we describe the design and construction of the CC-Activity-Based Protein Profiling Probe CE-P (CC-ABPP CE-P, Figure 1) and its subsequent use in identifying the targets of CE. An alkynyl group was introduced at the C-28 carboxylic moiety of the saponin scaffold, which enabled the hydrophilic PEG chain to link to biotin through a Cu(I)-catalyzed Huisgen 1,3-dipolar cycloaddition reaction.

**Scheme 1:**
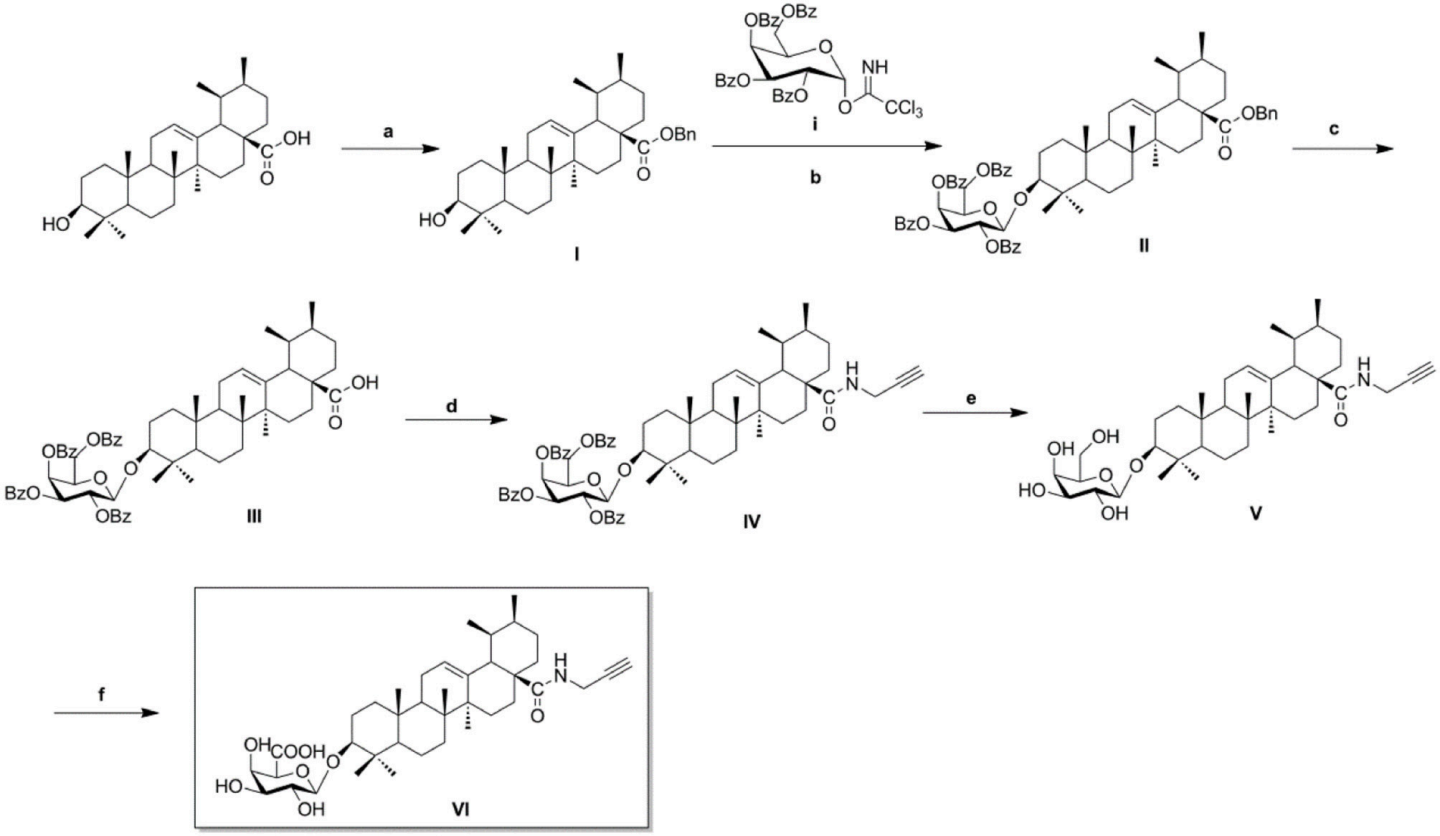
Synthesis of biotinylated probe CE-P. Reagents and conditions: (a) BnBr, K_2_CO_3_, TBAB, DCM–H_2_O, rt, 18 h; (b) glycosyl donor **i**, TMSOTf, 4Å MS, DCM, rt, 2–4 h; (c) H_2_, Pd–C (10%), EtOAc, reflux, 4–6 h; (d) HOBt, EDCI, propargylamine, rt, 6–8 h; (e) NaOMe, MeOH, rt, 2–3 h; (f), KBr, TEMPO, TBAB, Na_2_CO_3_/NaHCO_3_, Ca(ClO)_2_, 0^o^C, 8 h.

As illustrated in Scheme 1, naturally abundant ursolic acid was treated with benzyl bromide (BnBr), a potassium carbonate solution (K_2_CO_3_), and tetrabutylammonium bromide (TBAB) in dry dichloromethane (DCM) to obtain a good yield of compound **I**. The glycosyl donor **i** was prepared from galactose using the conditions reported by Schmidt (Schmidt and Michel, 1980). Compound **I** was reacted with glycosyl donor **i** under Lewis acidic conditions in the presence of trimethylsilyl trifluoromethanesulfonate (TMSOTf) to produce compound **II**, which was subjected to hydrogenation to obtain compound **III** in the presence of a catalytic amount of 10% Pd-C at atmospheric pressure. The above reaction conditions were reported in our previous paper. Compound **IV** was attained via amidation of the C-28 carboxyl group of saponin scaffold with propargylamine, followed by deprotection of the glycosyl groups in the presence of a NaOMe/MeOH solution to obtain compound **V**. In the final step, an oxidation reaction was performed using compound **V** and TEMPO/Ca(ClO)_2_ in the presence of KBr and a TBAB catalyst in an Na_2_CO_3_/NaHCO_3_ solution, yielding the CC-ABPP **CE-P** (compound **VI**).

### CE-P protects against ox-LDL-induced endothelial cell injury

**Figure 2:**
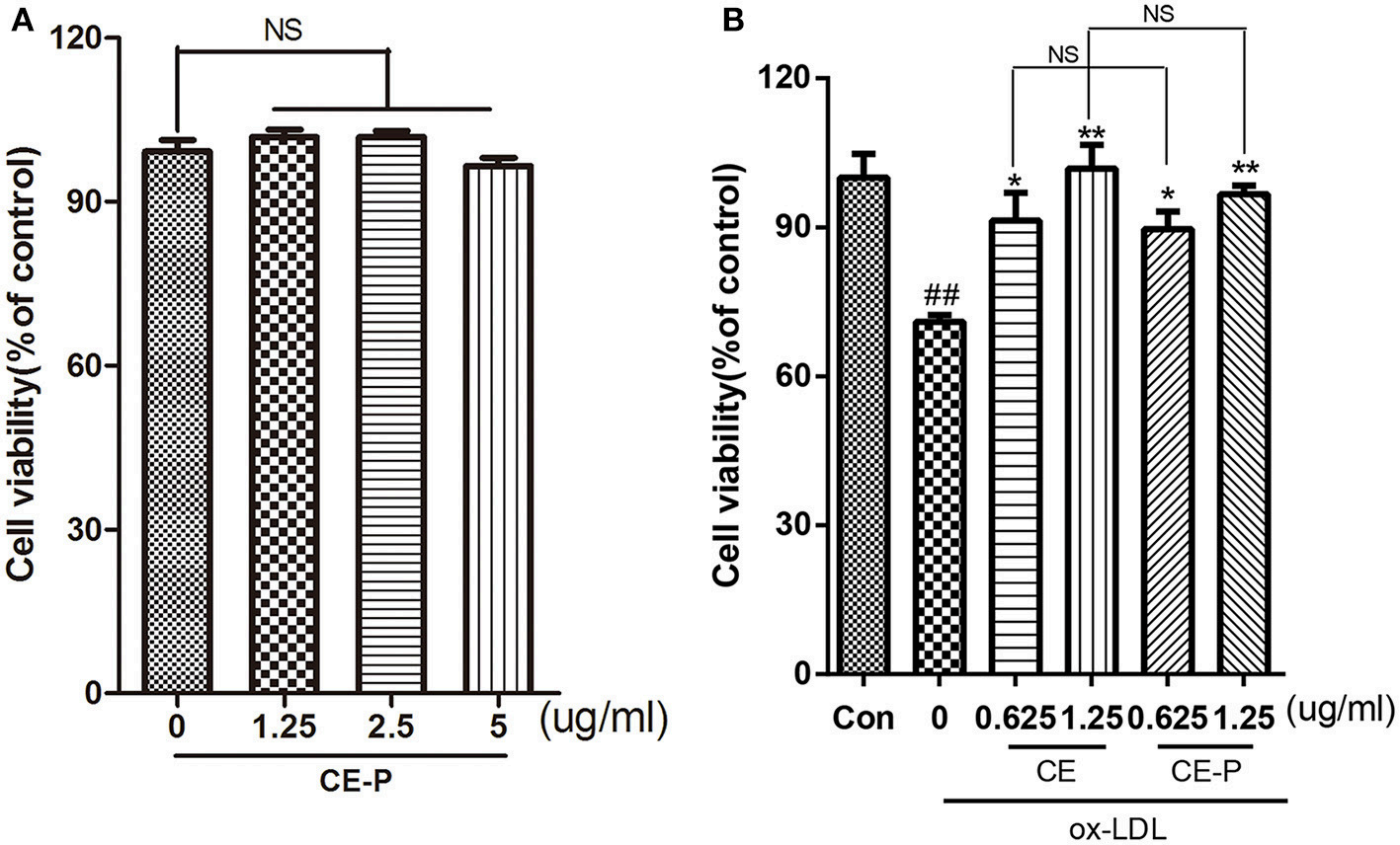
CE-P protects ox-LDL-induced endothelial cell injury. **(A)** To evaluated the cytotoxicity of CE-P, HUVECs were treated with CE-P alone (1.25, 2.5, 5 μg/mL) for 24 h and then the cell viability was measured by MTT assay. **(B)** HUVECs were pretreated with CE or CE-P (0.625, 1.25 μg/mL) for 8 h, then were incubated with or without ox-LDL for another 24 h and finally cell viability was assayed by MTT. The data are expressed as means ± *SD*. from three independent experiments. ^##^*P* < 0.01 vs. control group, *P < 0.05, **P < 0.01 versus ox-LDL treatment group. NS is no significance.

As shown in our previous study, CE protected against ox-LDL-induced endothelial cell injury (Tian et al., 2017b). In this context, we introduced a very small alkyne group into CE to create a click chemistry activity-based probe. We first measured cell viability using the MTT assay to investigate the activity of CE-P. The cytotoxicity of CE-P was measured, and the results shown in Figure 2A did not reveal obvious changes in cell viability. Then, we determined whether CE-P protects cells from ox-LDL-induced injury. As shown in Figure 2B, the control group was pretreated for 8h with vehicle then exposed without ox-LDL, the other groups exposed to ox-LDL exhibited dramatically decreased cell viability, whereas pretreatment with CE or CE-P (0.625 or 1.25 μg/mL) for 8 h significantly ameliorated cell injury. We found that there were no significant differences between the two compounds at the same doses for sustaining the cell viability. CE-P retained the ability of inhibiting ox-LDL induced HUVECs damage, and the presence of the small alkyne moiety does not affect the biological activity of CE.

### CE-P attenuates ox-LDL-induced HUVEC apoptosis

**Figure 3:**
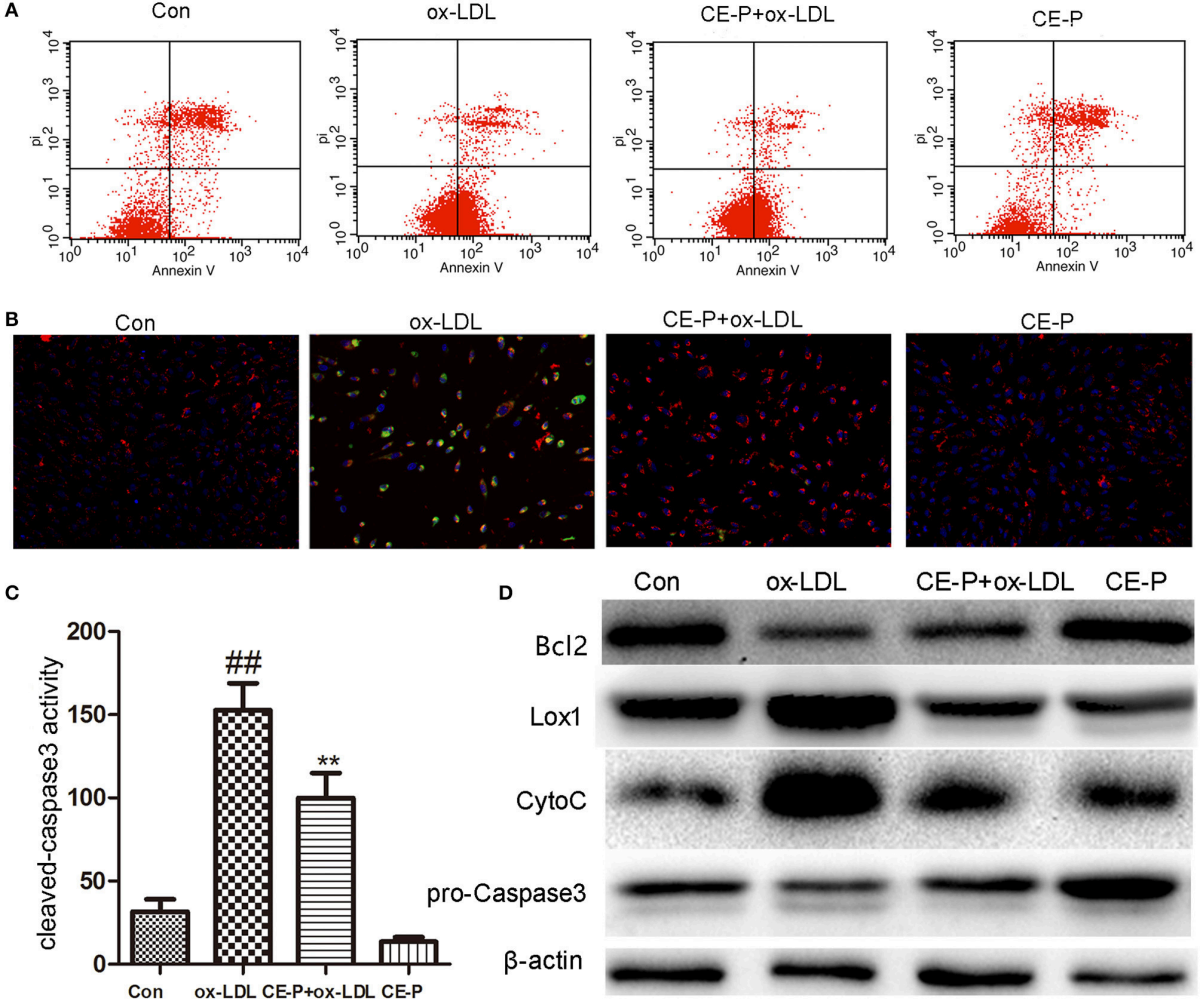
CE-P attenuates the ox-LDL induced HUVECs apoptosis. The protective effect of CE-P on ox-LDL-induced apoptosis was determined via AnnexinV/PI double staining, JC-1 staining, cleaved-caspase3 activity, and western blot assay. HUVECs were pretreated with CE-P (1.25 μg/mL) for 8 h and then incubated with or without ox-LDL for additional 24 h for associated measures. **(A)** After cell treatment, cell early apoptosis was measured via AnnexinV/PI double staining by flow cytometry. **(B)** The mitochondria damage during apoptosis was detected by JC-1 staining through fluorescence microscope. **(C)** At the final stage of apoptosis, the cleaved caspase3 activity was measured by fluorometric assay. **(D)** Apoptosis associated proteins Bcl2, Caspase3, Cytochrome C were evaluated by western blot. The data are expressed as means ± SD from three independent experiments. ^##^*P* < 0.01 vs. control group, **P < 0.01 vs. ox-LDL treatment group.

CE has been shown to protect against cell apoptosis (Tian et al., 2017b). We first detected the phosphatidylserine (PS) levels using Annexin V/propidium iodide (PI) double staining and flow cytometry to explore whether the effects of CE-P on protecting cells from ox-LDL-induced injury involved the inhibition of cell apoptosis. During the early stage of apoptosis, phosphatidylserine is exposed on the extracellular side of the cell membrane, and Annexin V specifically binds PS (Qin et al., 2015). As shown in Figure 3A, the protective effect of CE-P on ox-LDL-induced cell death following PS exposure was investigated using Annexin V/PI double staining and flow cytometry. An 8 h CE-P pretreatment decreased the percentage of Annexin V(+)/PI(–) cells. Mitochondrial damage is closely related to cell apoptosis, and a change in the mitochondrial membrane potential (ΔΨm) is one of the main functional markers of mitochondrial injury (Yu et al., 2016). JC-1 is an indicator of the mitochondrial transmembrane potential. As indicated by the JC-1 staining shown in Figure 3B, red fluorescence represents the normal mitochondria, and green fluorescence indicates HUVECs in which the mitochondrial membrane potential was depolarized. The ox-LDL-treated group exhibited a decrease in the intensity of red fluorescence and an increase of green signal. In contrast, the CE-P-pretreated group reversed this change by decreasing the green signal and increasing red fluorescence intensity, indicating that CE-P mitigated ΔΨm. Caspase-3, one of the critical enzymes involved in apoptosis, the active form cleaved capase-3 is induced at the late stage of apoptosis. DEVD-AFC is used to detect cleaved caspase-3 activity (Sun et al., 2014). As shown in Figure 3C, the CE-P pretreatment remarkably reduced cleaved caspase-3 activation. We evaluated the expression of apoptosis-related proteins using western blot analyses to further confirm the anti-apoptotic effects of CE-P on HUVECs. As shown in Figure 3D, CE-P increased the levels of Bcl2 and pro-caspase-3 and decreased the levels of Cytochrome C, consistent with our previous results showing the anti-apoptosis activity of CE. Lox-1 is the main ox-LDL receptor in HUVECs, and ox-LDL has been shown to induce the Lox-1 expression, which triggers cell apoptosis (Li et al., 2003; Li and Mehta, 2009). In our study, the CE-P pretreatment remarkably attenuated Lox-1 expression during ox-LDL-induced injury. Based on these results, CE-P protected HUVECs from ox-LDL-induced cell apoptosis.

### Profiling of CE-P target proteins in HUVEC cell lysates using click chemistry

**Figure 4:**
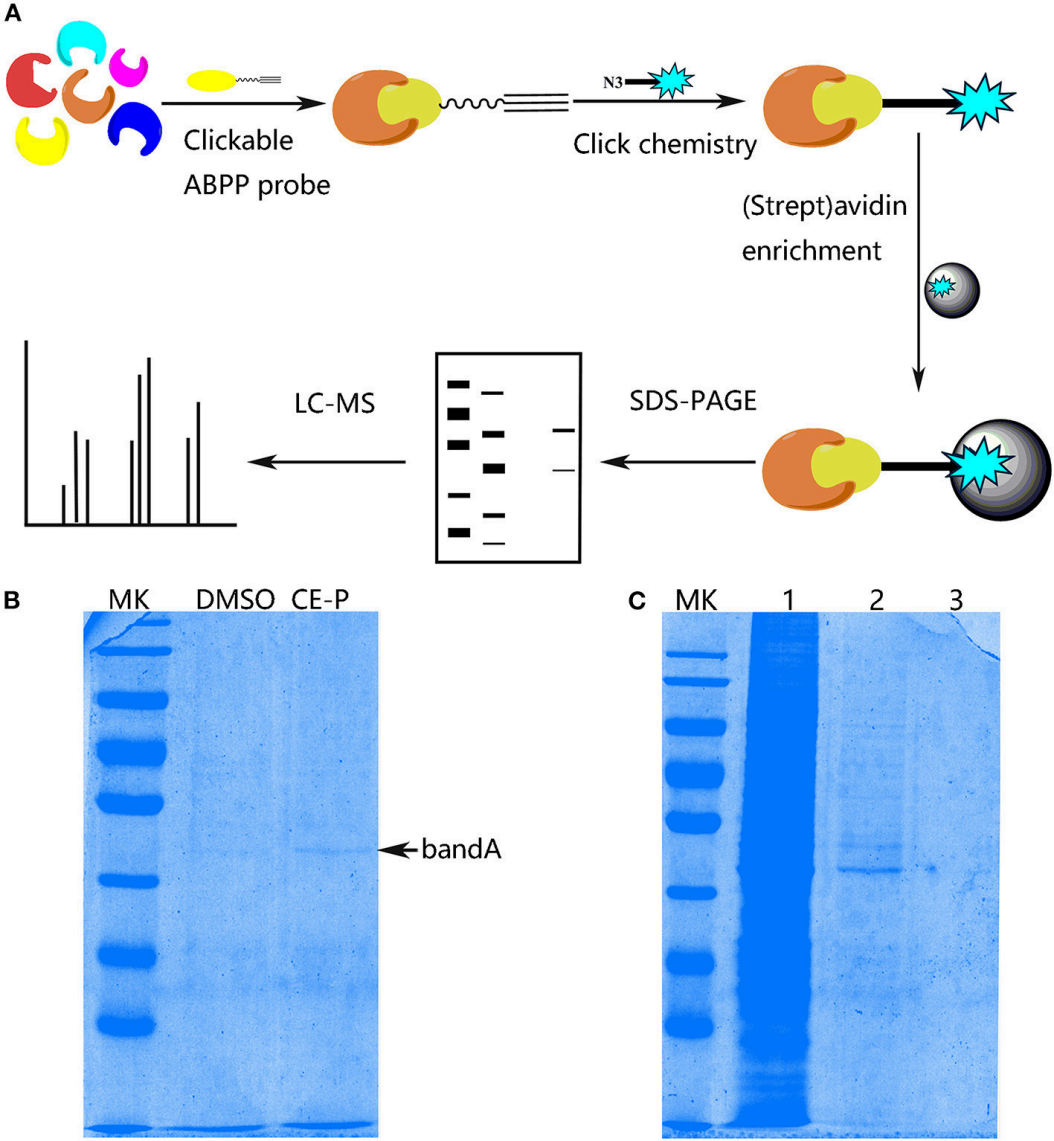
Protein Profiling of CE-P by click chemistry in HUVEC cell lysate. **(A)** Schematic image of proteome profiling of potential cellular targets of CE-P in HUVEC cell lysate. **(B)** The binding proteins was separated by SDS-PAGE and stained by coomassie blue staining. **(C)** The washing solution of CE-P was assayed by coomassie blue staining. Lane 1 is the whole cell lysate, lane 2 is the first washing solution, and lane 3 is the final washing solution.

With the effective chemical probe in hands, we performed pull-down experiments followed by proteomics analysis to identify the cellular targets of CE (Figure 4A). CE-P was first incubated with a cell lysate to identify the potential targets of CE. Proteomes were obtained from lysates incubated with DMSO and CE-P with a biotin-azide linker using a click reaction, after which the labeled proteins were enriched by an affinity pull-down method using streptavidin beads. The enriched proteomes were eluted and separated by one-dimensional gel electrophoresis (1DGE). As shown in Figure 4B, we observed a single labeled protein band in the cell lysate in the CE-P lane (band A, indicated by an arrow). We also examined the washes from the CE-P reaction to exclude non-specific binding of CE-P. After extensive washing with the binding buffer, the unbound proteins were eluted. In Figure 4C, lane 1 is the cell lysate, lane 2 is the first elution solution, and lane 3 is the final washing solution. Thus, band A represents proteins that specifically bound to CE-P (Yi et al., 2012). Next, we cut band A from the DMSO lane and CE-P lane for the liquid chromatography/tandem mass spectrometry (LC-MS/MS) analysis. The Mascot search algorithm was used to identify proteins from the resulting peptides identified by LC-MS/MS. A large number of proteins were identified from each LC/MS run. The proteins which got the scores > 100, were considered as reliable hits (Table S1) (Weerapana et al., 2010; Shi et al., 2011). Some of these proteins were inevitably non-specific proteins, many of which were “sticky” and/or highly abundant proteins. These proteins were automatically removed. “False” hits that appeared in control pull-down/LC/MS experiments were also eliminated to generate the final complete list of proteins (Table S1). Consequently, we identified 37 proteins as specific targets of CE-P.

### Hsp90AB1 as a potential target of CE-P

**Figure 5:**
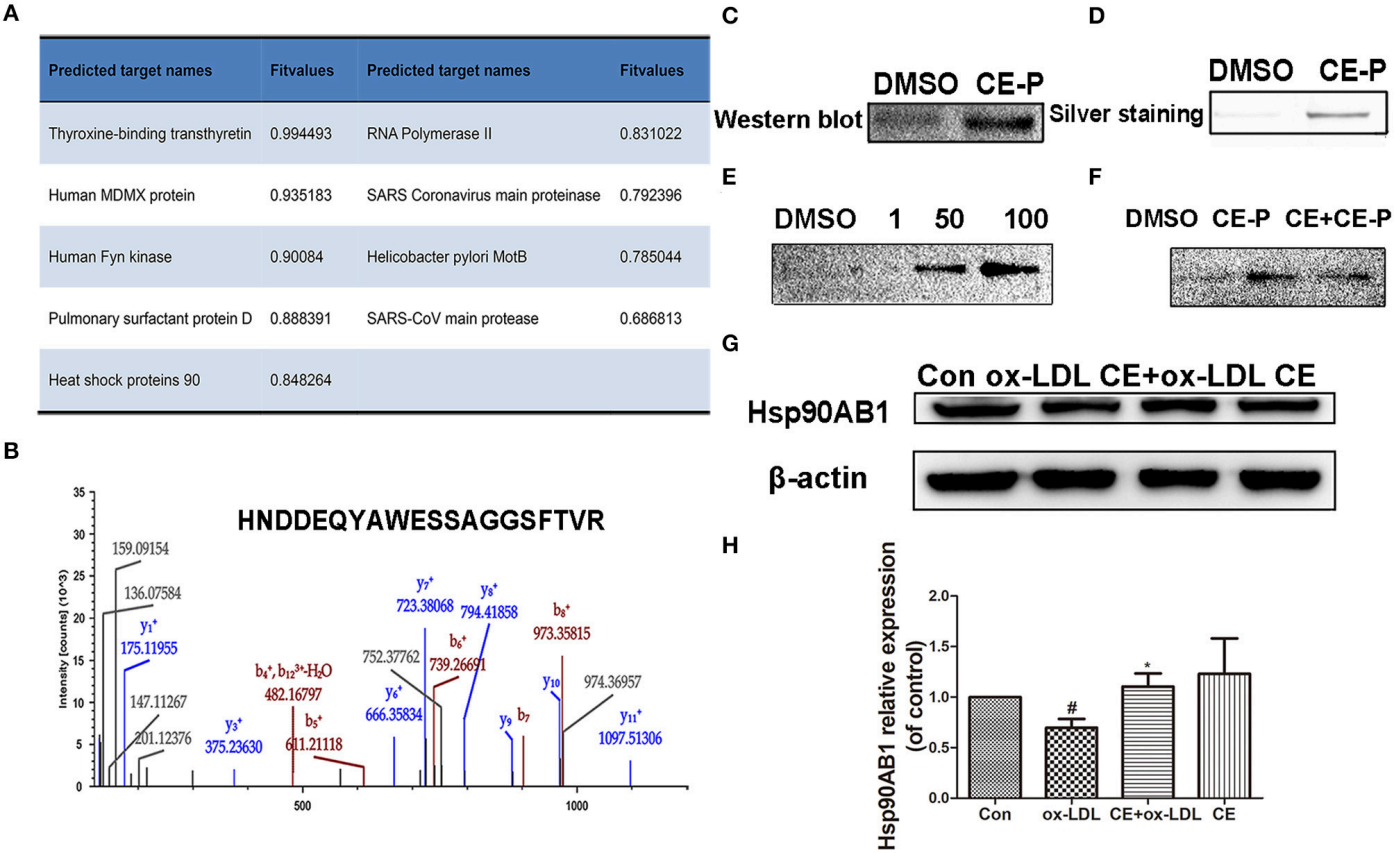
Hsp90ab1 as potential target of CE-P. **(A)** The predicted proteins by Discovery Studio 2016 software. **(B)** Identified peptide of Hsp90ab1 by LC/MS **(C)** Western-blotting validation of the CE-P target Hsp90ab1 by whole cell lysate pull-down assay. **(D)** The pull-down assay of the recombinant Hsp90AB1 by CE-P. **(E)** CE-P could pull down Hsp90AB1 in dose-dependent manner. **(F)** CE could inhibit the binding to Hsp90AB1 and then the proteins bound to CE-P were detected by Western blot. **(G)** Effects of CE on Hsp90AB1 expression levels in ox-LDL induced HUVEC damage. Cell lysates were harvested, and Western blot analysis was performed. β-actin expression was examined as the protein loading control. **(H)** Densitometric analysis was used to quantify the levels of Hsp90AB1. Values are expressed as the mean ± SD ^#^*p* < 0.05 ox-LDL group vs. control group; *p < 0.05, vs. ox-LDL group.

The molecular targets of CE-P were predicted using Discovery Studio 2016 software. Nineteen potential targets were found and shown to have probable relationships with the pharmacological effects of CE-P. Among these candidates, we selected targets with scores > 0.5 for the subsequent investigations and finally identified 9 proteins, as shown in Figure 5A. Moreover, Hsp90 which was predicted with a higher score 0.848264, was also identified by gel proteomic with the high score 217 in Table S1 and Figure 5B. Comparing these above results we thought Hsp90AB1 might be one of potential targets and be critical for cell apoptosis (Cohen-Saidon et al., 2006; Lanneau et al., 2007; Didelot et al., 2008; Chen et al., 2009). To further validate Hsp90AB1 as the direct binding target of CE-P, we confirmed the identity of the proteins that were pulled down using immunoblotting with their respective antibodies. As shown in Figure 5C, the CE-P pull-down precipitated Hsp90AB1, but almost no signal was observed in the control group. To verify the interaction of CE-P with Hsp90AB1, we incubated recombinant Hsp90AB1 protein with CE-P. As shown in Figure 5D, Hsp90AB1 was obviously pulled down by CE-P, which was detected by silver staining. We also found that CE-P can pull down Hsp90AB1 in dose-dependent manner as shown in Figure 5E. We incubated HUVEC cell lysates with CE-P in the absence or the presence of an excess amount of CE for competitive binding. As shown in Figure 5F, Hsp90AB1 was obviously pulled down by CE-P, moreover, an excess amount of CE effectively blocked the binding of Hsp90AB1 to CE-P, which were detected by Western blot. Taken together, the above results unequivocally confirmed a direct interaction between CE-P and Hsp90AB1. To further investigate the potential biological role of CE about Hsp90AB1, we then detected the effects of CE on Hsp90AB1 expression levels in ox-LDL induced HUVEC damage. Figures 5G,H showed that CE pretreatment significantly inhibited the down-regulation of the ox-LDL-induced Hsp90AB1 expression.

### Molecular docking between CE/CE-P and Hsp90AB1

**Figure 6:**
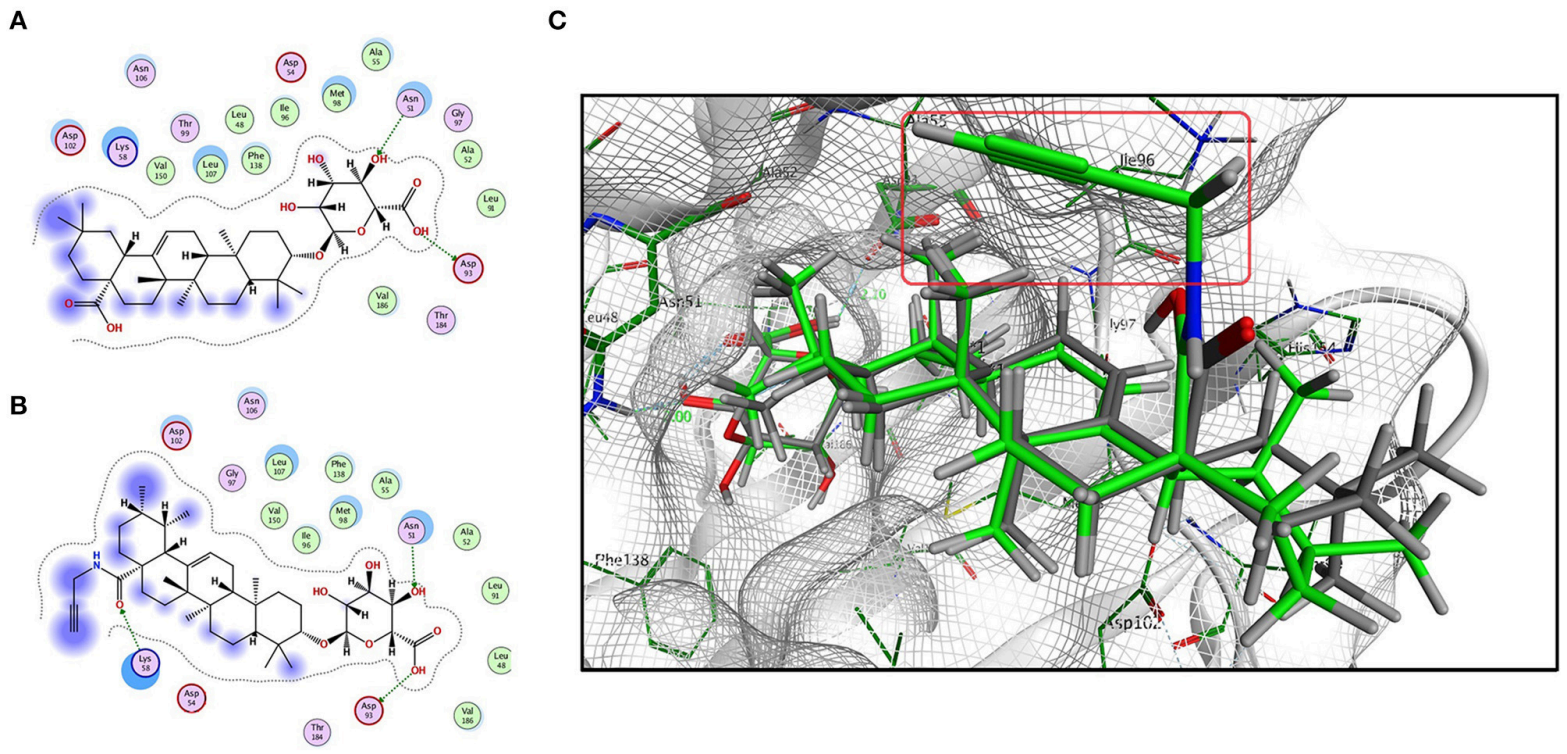
Modeling study of the structure of CE/CE-P binding to Hsp90AB1 protein. **(A)** Two-dimensional ligand interaction diagram of CE and Hsp90AB1. **(B)** Two-dimensional ligand interaction diagram of CE-P and Hsp90AB1. **(C)** Three-dimensional modeling of CE/CE-P binding with Hsp90AB1.

Based on the predicted molecular targets, we analyzed the possible interaction between CE/CE-P and the 3D Hsp90AB1 receptor binding sites (PDBID: 3NMQ) using Molecular Operating Environment (MOE) software package. The *S*-values (CE: −8.70 and CE-P: − 8.78) were obtained based on the virtual calculation of the interaction of CE/CE-P with the targeted Hsp90 AB1 protein. Molecular modeling of CE/CE-P showed that both two compounds could bind to the N-terminal domain of Hsp90AB1 and participated in important hydrogen bonds with key amino acid residues Asp 93 and Asn 51 (Figures 6A,B). As shown in Figure 6C, the glycosyl moieties of CE (gray) and CE-P (green) are responsible important for binding with the key amino acid residuces of Hsp90AB1 with amino acid residues, and the propargyl group (red frame) that exposing on the edge of the pockets were designed for “clicking” conveniently with biotin tag.

### SPR analysis of CE/CE-P binding to Hsp90AB1

**Figure 7:**
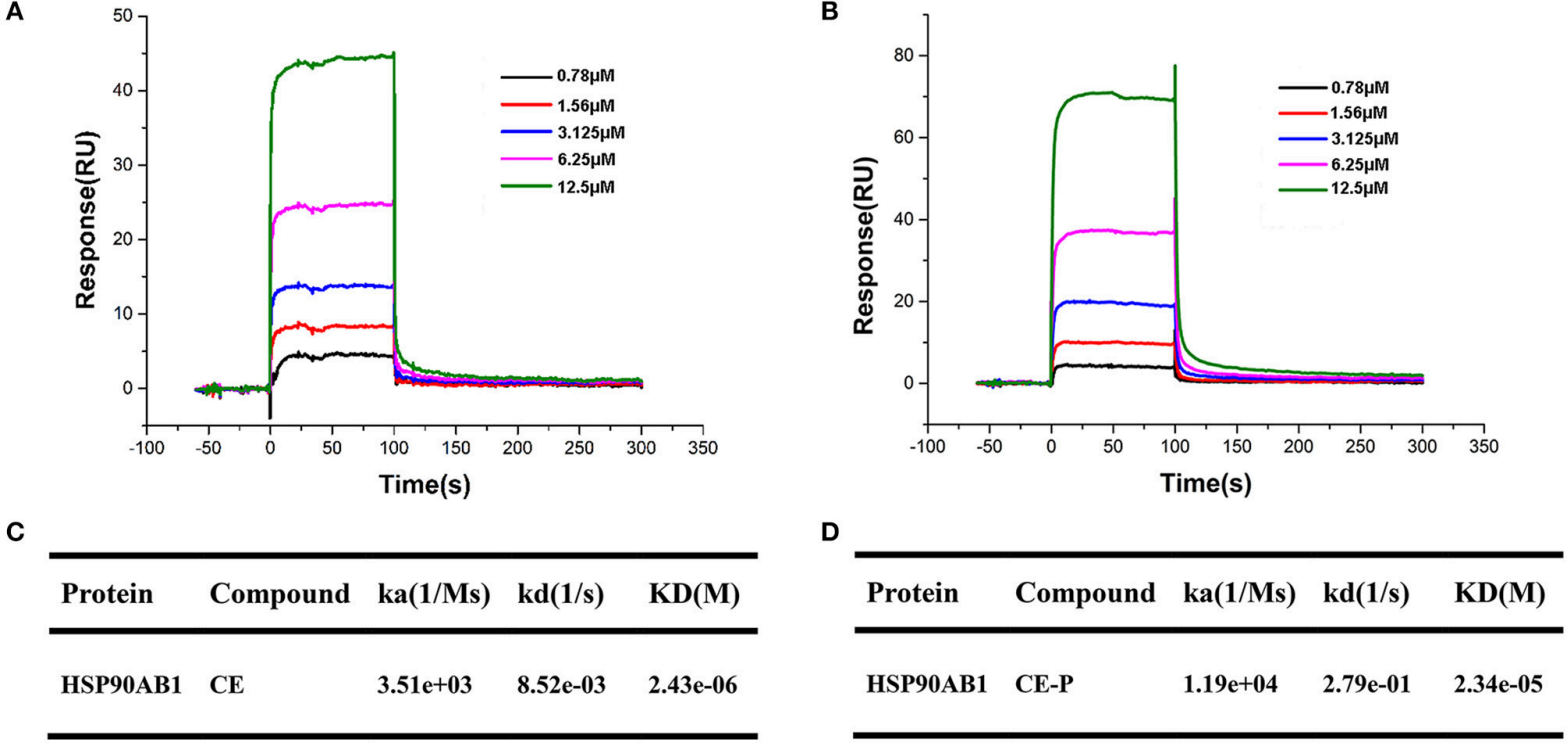
SPR analyses of CE or CE-P binding to Hsp90AB1. Hsp90AB1 immobilized to a CM5 Sensor Chip was provided with the CE/CE-P at concentrations varying from 0.75 to 12.5 μM. **(A,B)** Representative binding curves of CE **(A)** and CE-P **(B)** binding to Hsp90AB1. **(C,D)** Kinetic binding constants of CE **(C)**/CE-P **(D)** with Hsp90AB1.

Surface plasmon resonance (SPR) biosensors are most commonly applied for real-time dynamic analysis and measurement of interactions in bio-molecular studies and compounds analysis without the need for labeling processes. In our research, we applied this system to confirm the interaction of CE/CE-P with Hsp90AB1 and explore its binding affinity. As shown in Figures 7A,B, SPR data analysis revealed that both CE and CE-P could bind to Hsp90AB1 in a dose-dependent manner. The KD-value of CE-P binding to Hsp90AB1 was 23.4 μM (Figure 7D), and CE was 2.34 μM (Figure 7C).

## Discussion

The design and synthesis of potential probes represents a major challenge for target identification. In our previous study, the introduction of a substituent at the C-28 position of CE maintained its protective effects. Based on the results of preliminary SAR studies, amide derivatives of CE that containing ursane and galactoside scaffolds maintained similar activity to the parental compound CE. In the current study, the *N*-propargylamide derivative CE-P was chosen as the clickable activity-based probe in which the biotin tag was introduced using a Cu (I)-catalyzed Huisgen 1, 3-dipolar cycloaddition reaction. According to the results of the MTT assay, the CE-P probe exhibited promising protective effects against ox-LDL-induced HUVEC damage. We also confirmed that CE-P protects against apoptosis using Annexin V/PI staining, JC-1 staining, caspase-3 activity assays and western blotting. Based on these results, CE-P maintains its anti-apoptosis activity and is suitable for use in further research.

In this context, we introduced a very small alkyne group into CE to create a clickable activity-based probe. Unlike the bulky biotin tag, the small alkyne group does not affect the interaction of this compound with the potential targets *in vitro* or its ability to penetrate the plasma membrane. In our previous reports, we utilized an ABPP probe and identified ~750 potential targets, however, with this probe, we identified 37 proteins as the most promising targets using the gel-based strategy. The clickable probe excluded a significant number of non-specific proteins and increased the possibility of identifying potential targets to prevent further injury. The probe will also be used to explore potential targets *in vivo* in future studies.

The ability to predict and interpret the mechanisms of action and biological targets of drugs has become feasible with the development of computational chemistry. Using DS 2016 software, we screened 9 proteins as potential targets that modulate a number of biological functions. Among these candidates, we focused on Hsp90AB1 because it had higher scores both in DS vital prediction and proteomics identification of the pull-down targets with CE-P. To rule out the interference of others, we used Hsp90AB1 pure proteins to repeat the binding experiments. The SPR results also revealed the affinity characters between them. By affinity analysis, we found CE-P (23.4 μM) had a relatively weaker affinity than CE (2.43 μM), but still maintained the property to bind the Hsp90AB1 in a dose-dependent manner. To explore their mode of action, we performed virtual assay and found both ligands could bind with Hsp90AB1, maybe it was the way that CE could influence the target function. However, this binding site was speculative and based only on molecular modeling. To confirm its exact binding domain of CE with Hsp90AB1, it needs more powerful researches such as ATP/ADP site mutation and cocrystallization to prove this.

The Hsp90s are a family of molecular chaperones that function in the cellular stabilization, regulation, and activation of a range of “client” protein. The human isoforms of Hsp90 include Hsp90α and Hsp90β (also named Hsp90AA1 and Hsp90AB1) which are 85% identical (Li and Buchner, 2013; Synoradzki and Bieganowski, 2015). Their distinct functions have been identified (Lamoth et al., 2016). Hsp90α correlates with tumor invasiveness, angiogenesis and metastasis (Tsutsumi et al., 2008; Song et al., 2010). In contrast, Hsp90β appears to have specific role in the anti-apoptitic functions of Bcl2 and cIAP1 (Cohen-Saidon et al., 2006; Lanneau et al., 2007; Didelot et al., 2008; Chen et al., 2009). Hsp90α and Hsp90β were also recently found to have differing effects on the activity of endothelial nitric oxide synthase (Cortes-González et al., 2010; Fismen et al., 2012). In our research, the specific domains of Hsp90AB1 were identified by LC/MS of pull-down proteins. CE could protect ox-LDL induced apoptosis and this coincides with the function of Hsp90AB1, so we mainly focused on Hsp90AB1. Indeed, we also identified one non-specific sequence (HFSVEGQLEFR) of Hsp90AA1 and Hsp90AB1 except most of the specific sequences. Might it was also a possible insight for other Hsp90s such as Hsp90AA1 as the potential target of CE, but was still need a lot of experimental results to prove it. Hsp90AB1, as molecular chaperone, interact with a lot of clients to form complexes to regulate its activity. In our research, we have confirmed CE could directly bind to Hsp90AB1 by SPR assay, if CE binds Hsp90AB1 clients still need more exploration (Hartson and Matts, 2012).

Post-translation modification (PTM) is central to biology by expanding and modulating the function of a large number of proteins. PTM contains a lot of styles such as attachment of small moieties cofactors, phosphorylation, acetylation, methylation, ubiquitylation (Hartley et al., 2015). Hsp90 undergoes extensive post-translational modifications, such as posphorylation, acetylation, S-nitrosylation, and ubiquitination (Mollapour and Neckers, 2012). Each of these factors can impact significantly on protein structure and function thus influencing and even enabling inherent protein activity. In our research, we confirmed CE binds Hsp90AB1 to interfere its function. If there is some other post-translation modifications involved in their interaction need more exploration.

Taken together all these results, we have focused our attention on Hsp90AB1 as one potential target of CE in HUVECs for further studies. The other candidates in this report should still be considered as potential targets, but their roles must be confirmed in additional experiments. In our future studies, we will perform *in vivo* experiments to further examine all the candidates.

## Conclusion

In summary, our present researches employed chemical proteomics and click chemistry approaches for the first time to explore the targets of CE in HUVECs and identified Hsp90AB1 as possible molecular target. In our report, we designed and synthesized the clickable CE-P probe and showed that it exhibited similar activity to CE by inhibiting ox-LDL-induced cell injury. For the sake of fishing its targets, we pulled down the proteins in HUVECs cell lysate with CE-P and identified 37 potential targets using the gel-based proteomic strategy. Combining fishing data by DS 2016, we finally focused on Hsp90AB1 protein on account of the higher scores both in the pull-down assay and virtual assay. To confirm the target, we firstly detected its existence in whole cell lysate by western blotting. The probe CE-P performed the same mode of interaction and had the same binding site with Hsp90AB1, which were proved by the competitive inhibition experiment and molecular docking software respectively. To further confirm the interactions of CE-P with Hsp90AB1, we used the recombinant Hsp90AB1 protein to exclude the interference of others protein in cell lysate. Moreover, the SPR analysis revealed that both CE/CE-P could bind to Hsp90AB1 with the similar protein affinity which proved that both CE and CE-P could direct bind to protein Hsp90AB1. Based on upon reliable data, we believe that Hsp90AB1 is the potential target of CE, and will be a more promising target for future explorations.

## Author contributions

G-BS, X-DX, and X-BS conducted the study. SW and YT designed the detailed experiments, performed the study, and collected and analyzed data. J-YZ, H-BX, PZ, MW (sixth author), S-BL, YL and MW (ninth author) took part in the experiments in this study. All Authors commented the study and approved the final manuscript.

### Conflict of interest statement

The authors declare that the research was conducted in the absence of any commercial or financial relationships that could be construed as a potential conflict of interest.

## Abbreviations

CE: Calenduloside E
CE-P: Calenduloside E Probe
HUVEC: human umbilical vein endothelial cell
ABPP: Activity-based protein profiling
CC-ABPP: click chemistry-Activity-based protein profiling
SAR: structure-activity relationship
BnBr: benzyl bromide
K_2_CO_3_: potassium carbonate solution
TBAB: tetrabutylammonium bromide
DCM: dichloromethane
TMSOTf: trimethylsilyl trifluoromethanesulfonate
ox-LDL: Oxidized Low density lipoprotein
PI: propidium iodide
1DGE: one-dimensional gel electrophoresis
LC-MS/MS: liquid chromatography/tandem mass spectrometry
Hsp90: Heat shock protein.
