# Supplementary material for: Targets Fishing and Identification of Calenduloside E as Hsp90AB1: Design, Synthesis, and Evaluation of Clickable Activity-Based Probe
Source: Front Pharmacol. 2018 May 23;9:532. doi: 10.3389/fphar.2018.00532 (PMC5974765; doi:10.3389/fphar.2018.00532)
Supplement: Supplementary file 1 [file Data_Sheet_1.DOCX]

**Targets fishing and identification of Calenduloside E as Hsp90AB1: design, synthesis and evaluation of clickable activity-based probe**

**Shan Wang ^a,#^, Yu Tian ^a,#^, Jing-Yi Zhang^a^, Hui-Bo Xu^b^, Ping Zhou^a^, Min Wang^a^, Sen-Bao Lu^c^, Yun Luo^a^, Min Wang^d^, Gui-Bo Sun^a*^, Xu-Bong Xu ^a*^and Xiao-Bo Sun^a*^**

^a^Beijing Key Laboratory of Innovative Drug Discovery of Traditional Chinese Medicine (Natural Medicine) and Translational Medicine, Institute of Medicinal Plant Development, Chinese Academy of Medical Sciences & Peking Union Medical College, Beijing, 100193, P. R. China.

^b^Academy of Chinese Medical Sciences of Jilin Province, Changchun, Jilin, China.

^c^Department of Bioengineering, Santa Clara University, Santa Clara, California, USA
^d^Harbin University of Commerce, Harbin, 150076, Heilongjiang, P. R. China.

^#^These authors contributed equally to this work.
*Correspondence authors. Address: Institute of Medicinal Plant Development, Chinese Academy of Medical Sciences and Peking Union Medical College, No. 151, Malianwa North Road, Haidian District, Beijing 100193, PR China. Tel: +86-010-57833013; Fax: +86-010-57833013.
E-mail addresses: xdxu@implad.ac.cn (Xu-Dong Xu), gbsun@implad.ac.cn (Gui-bo Sun), sun_xiaobo163@163.com (Xiao-bo Sun).

1, **Design and Synthesis of the CC-Activity-Based Protein Profiling Probe CE-P based on CE.**

^
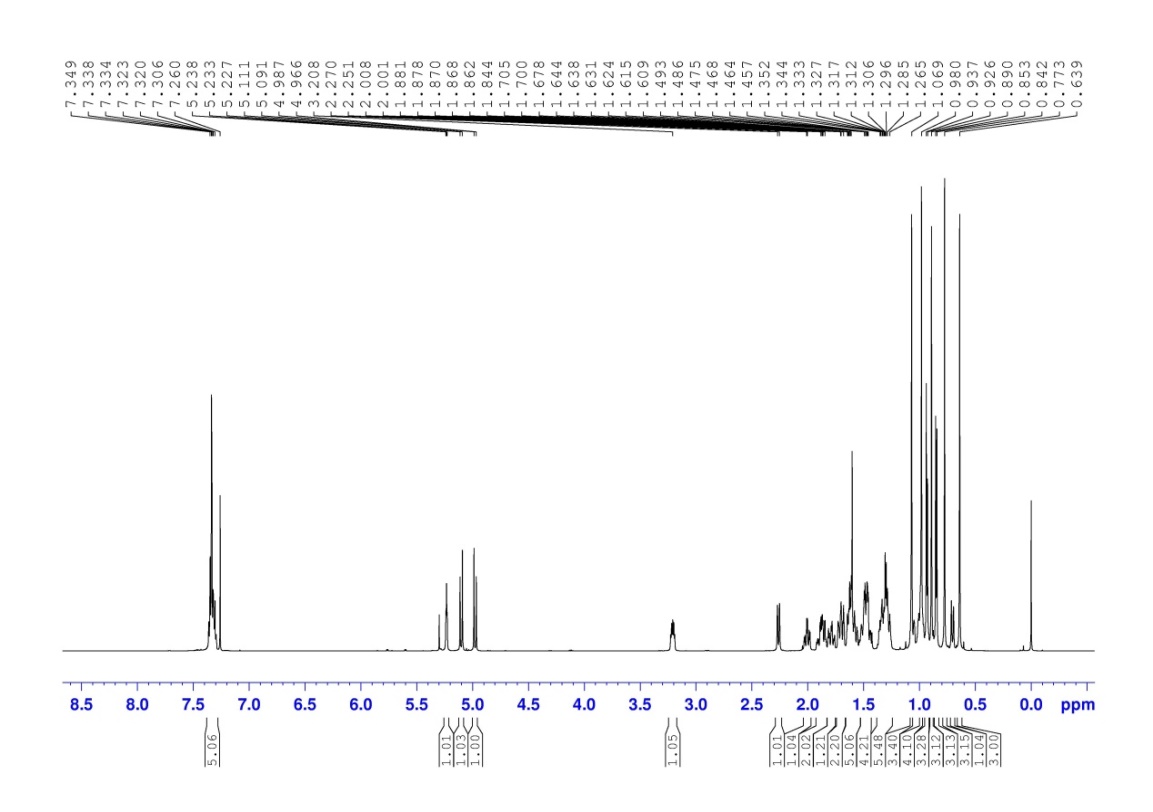
^

^1^H NMR of compound **I**

**
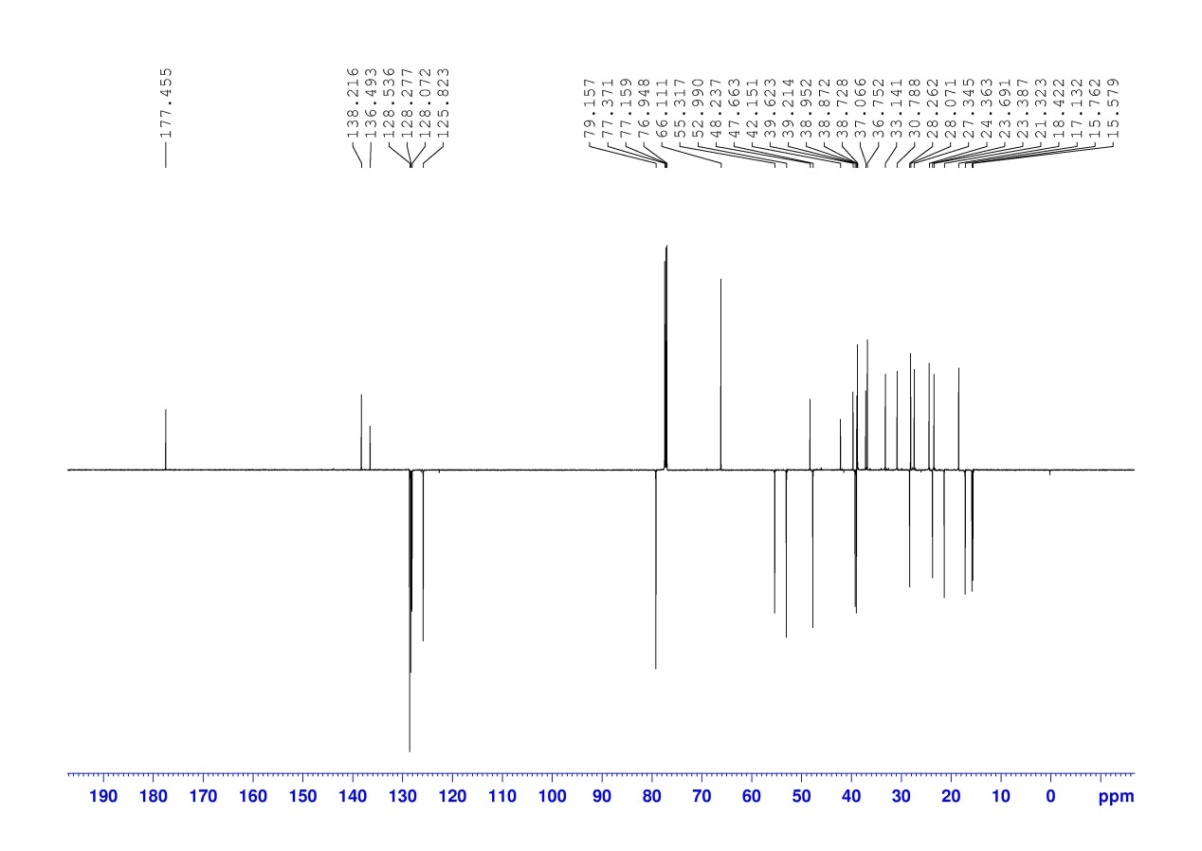
**

^13^C NMR of compound **I**

^
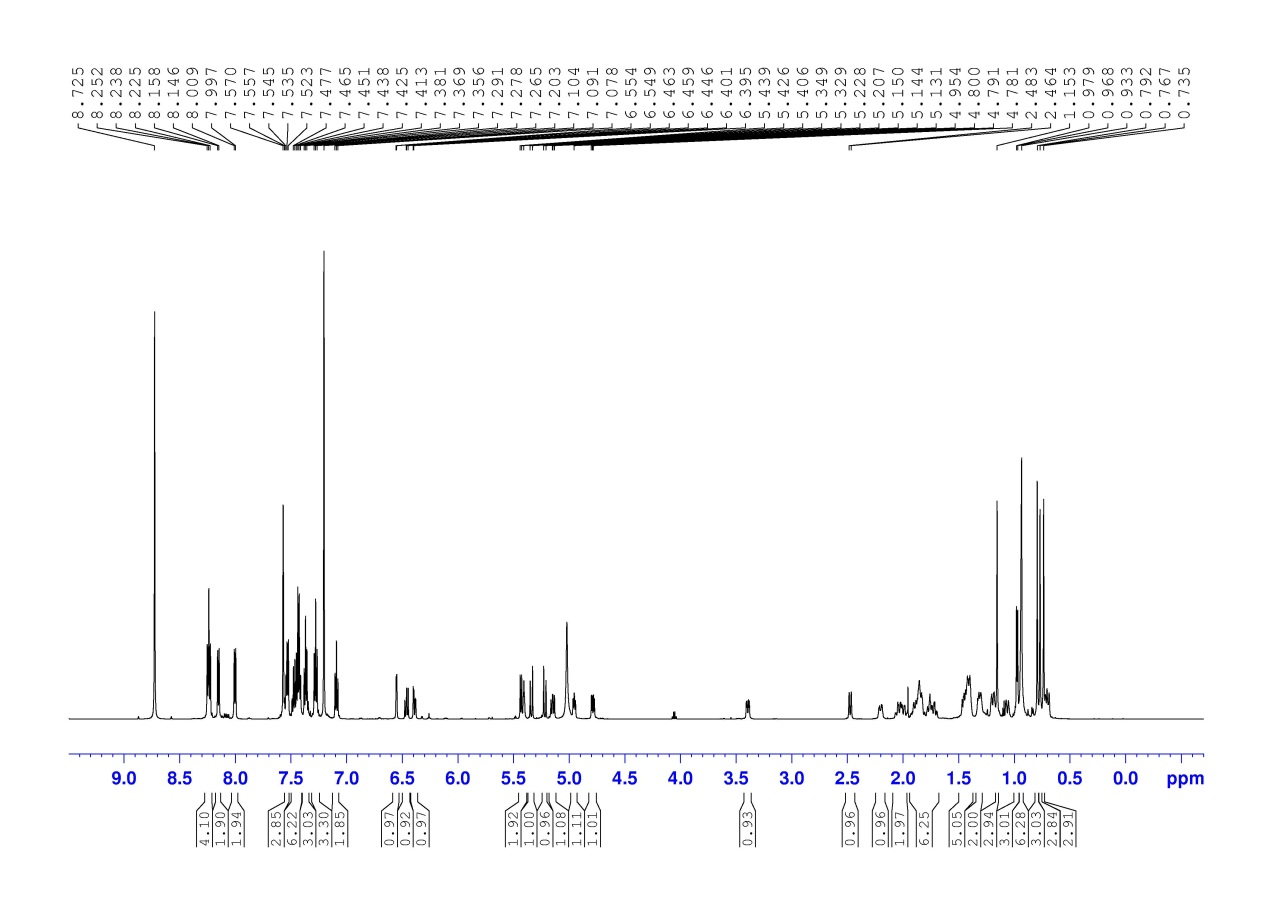
^

^1^H NMR of compound **II**

^
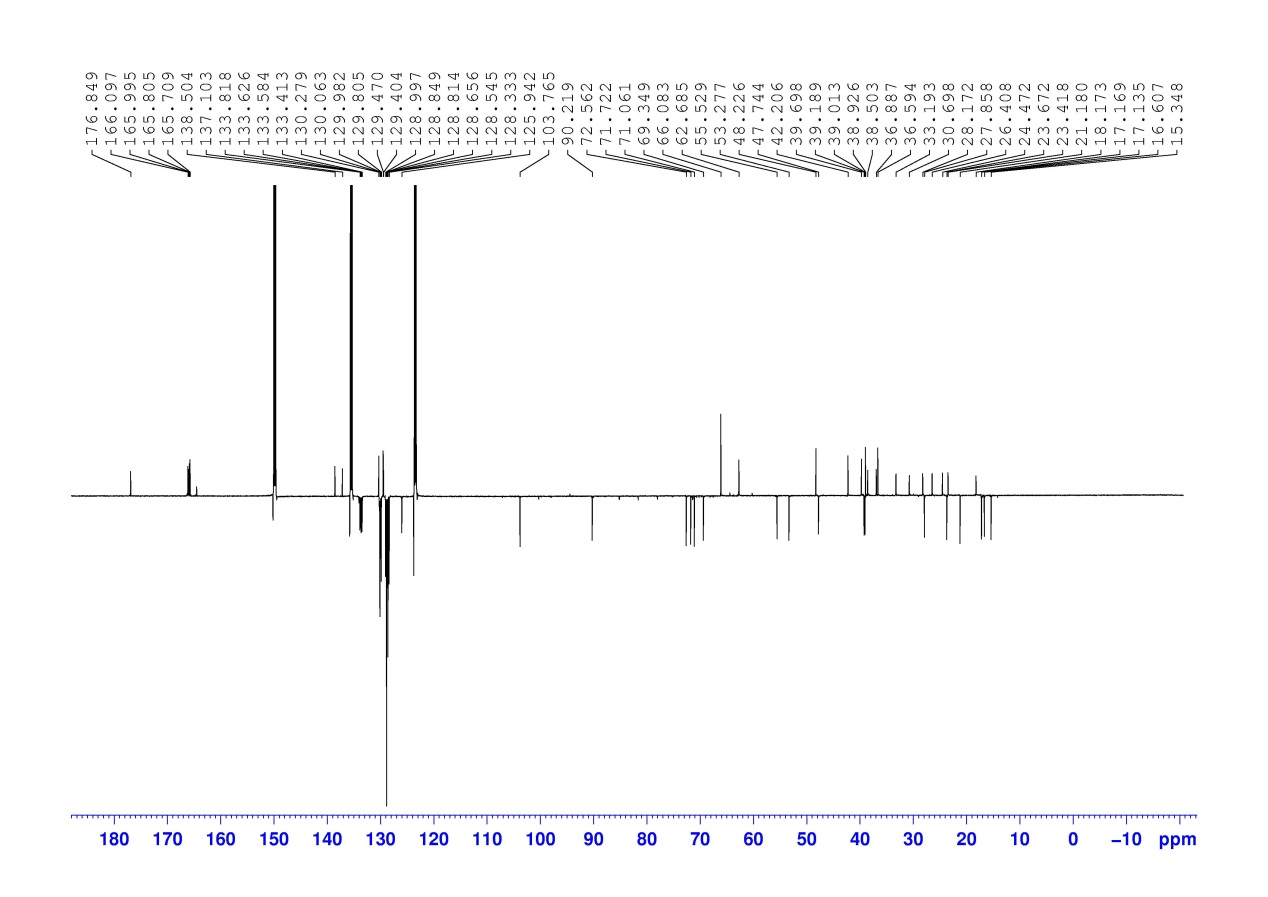
^

^13^C NMR of compound **II**

^
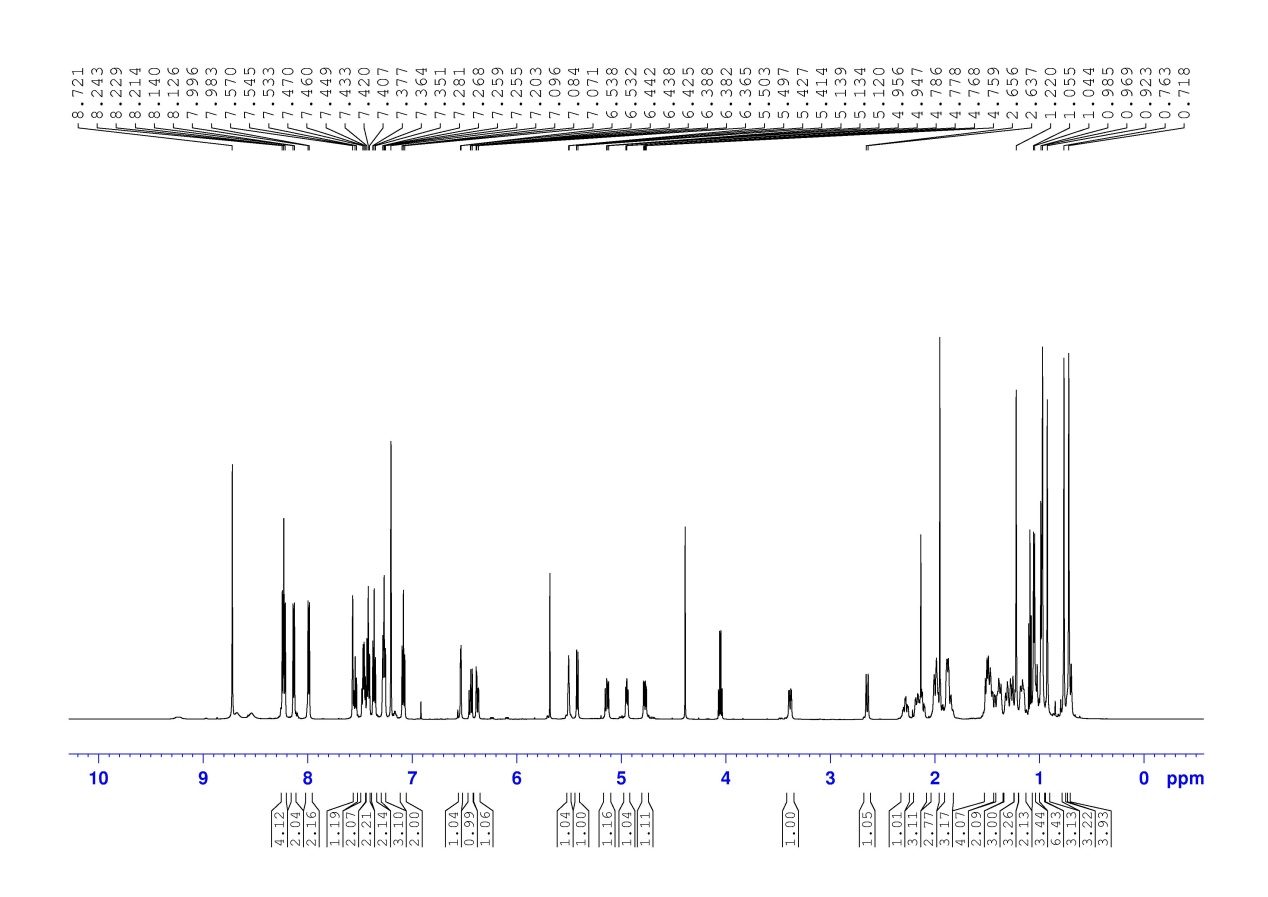
^

^1^H NMR of compound **III**

^
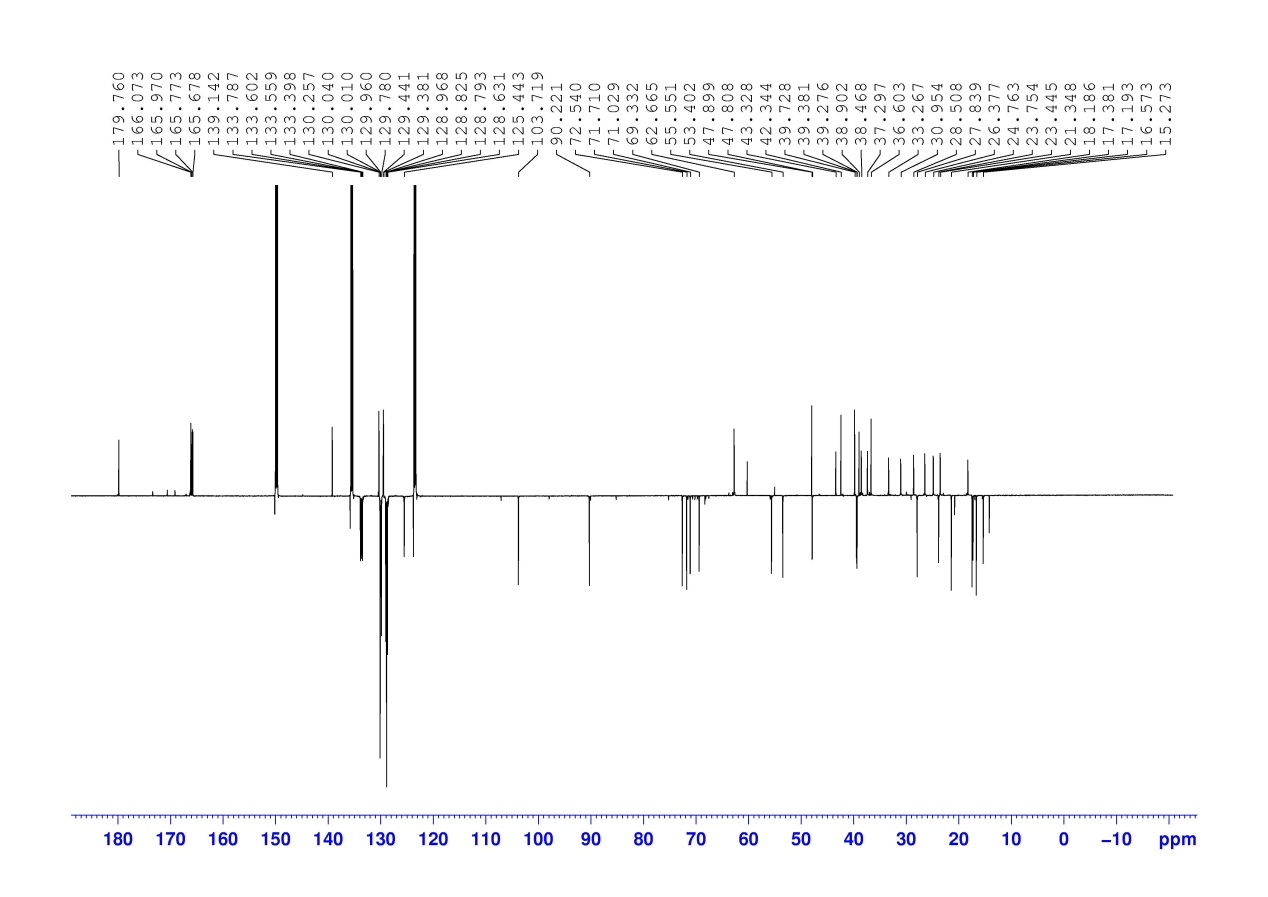
^

^13^C NMR of compound **III**

^
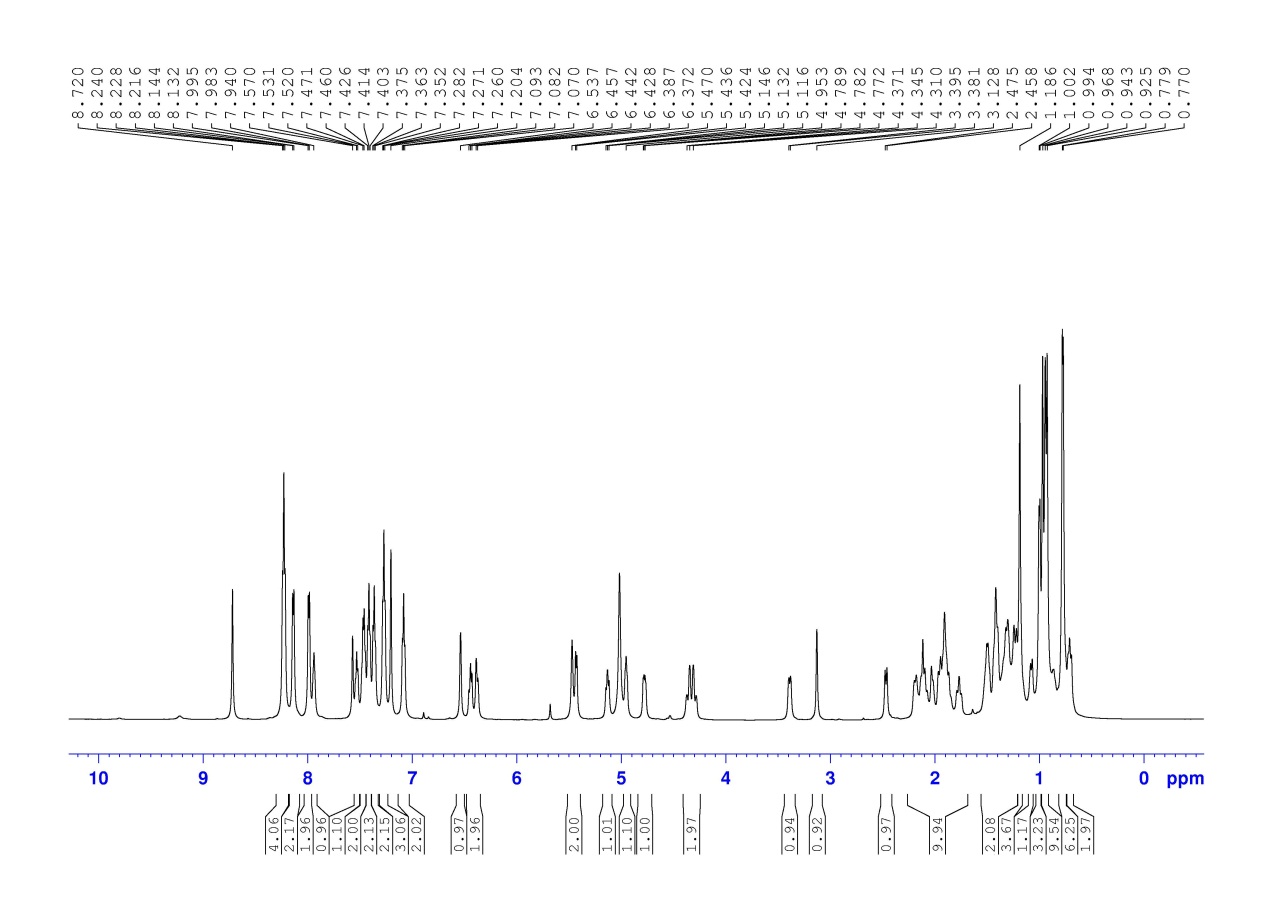
^

^1^H NMR of compound **IV**

^
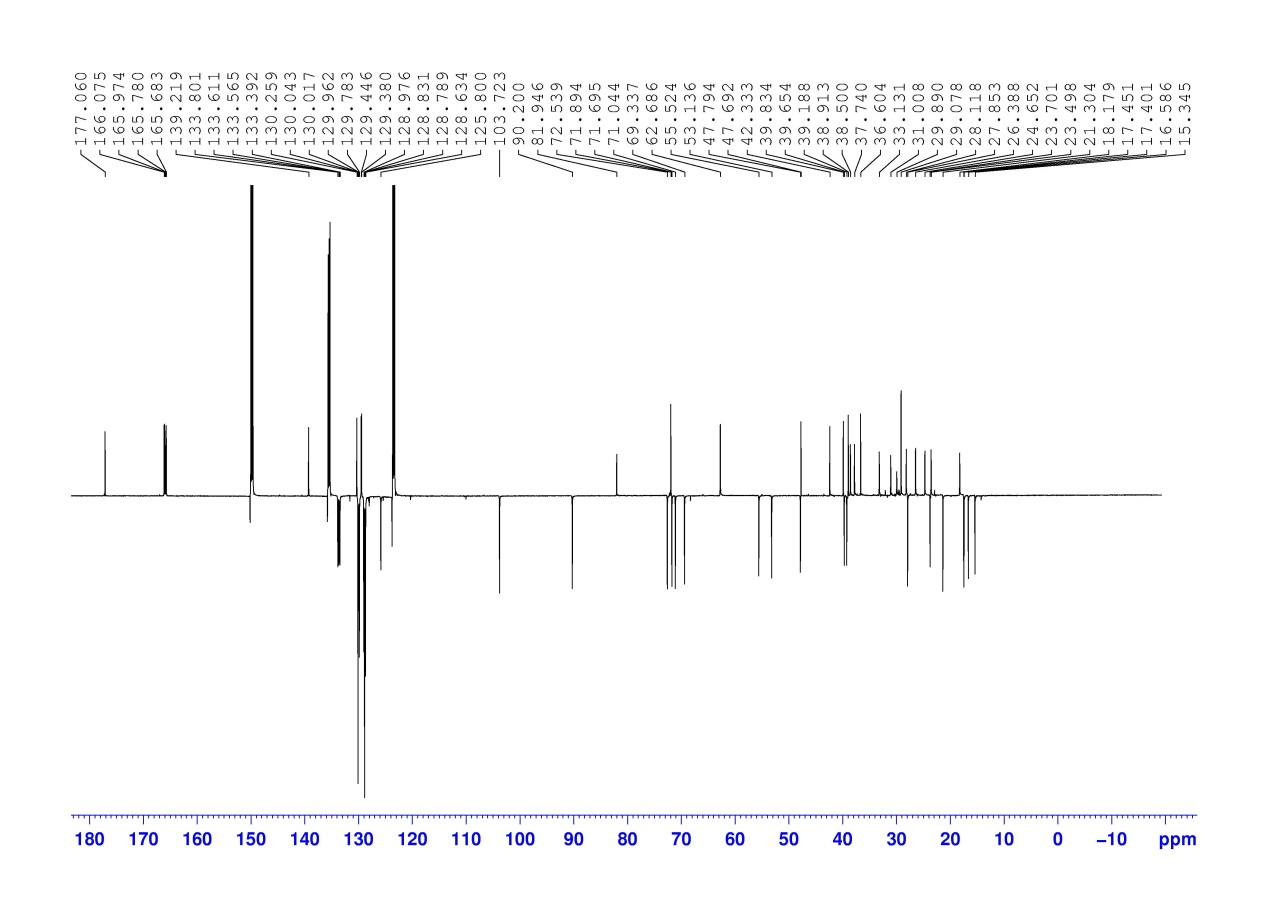
^

^13^C NMR of compound **IV**


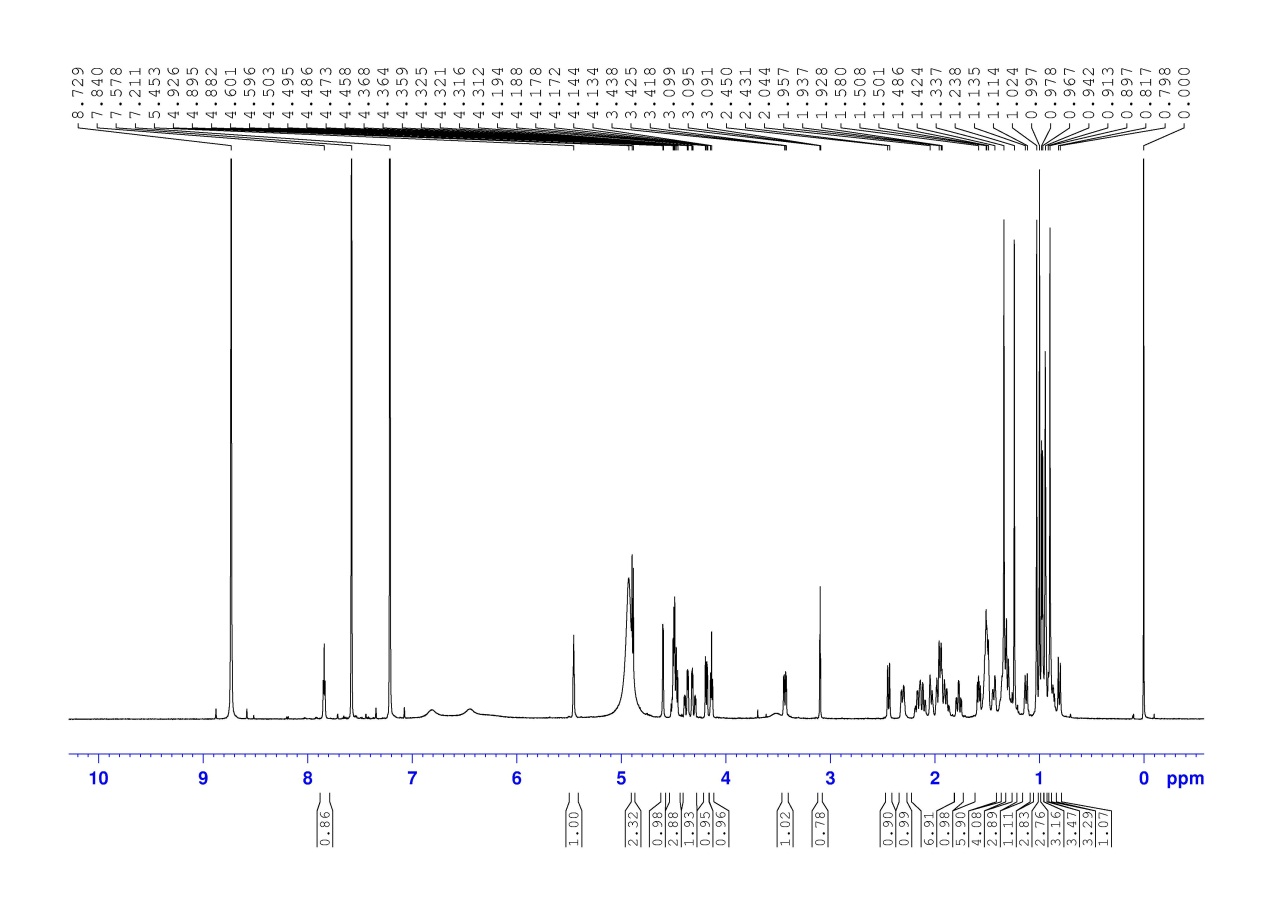


^1^H NMR of compound **V**


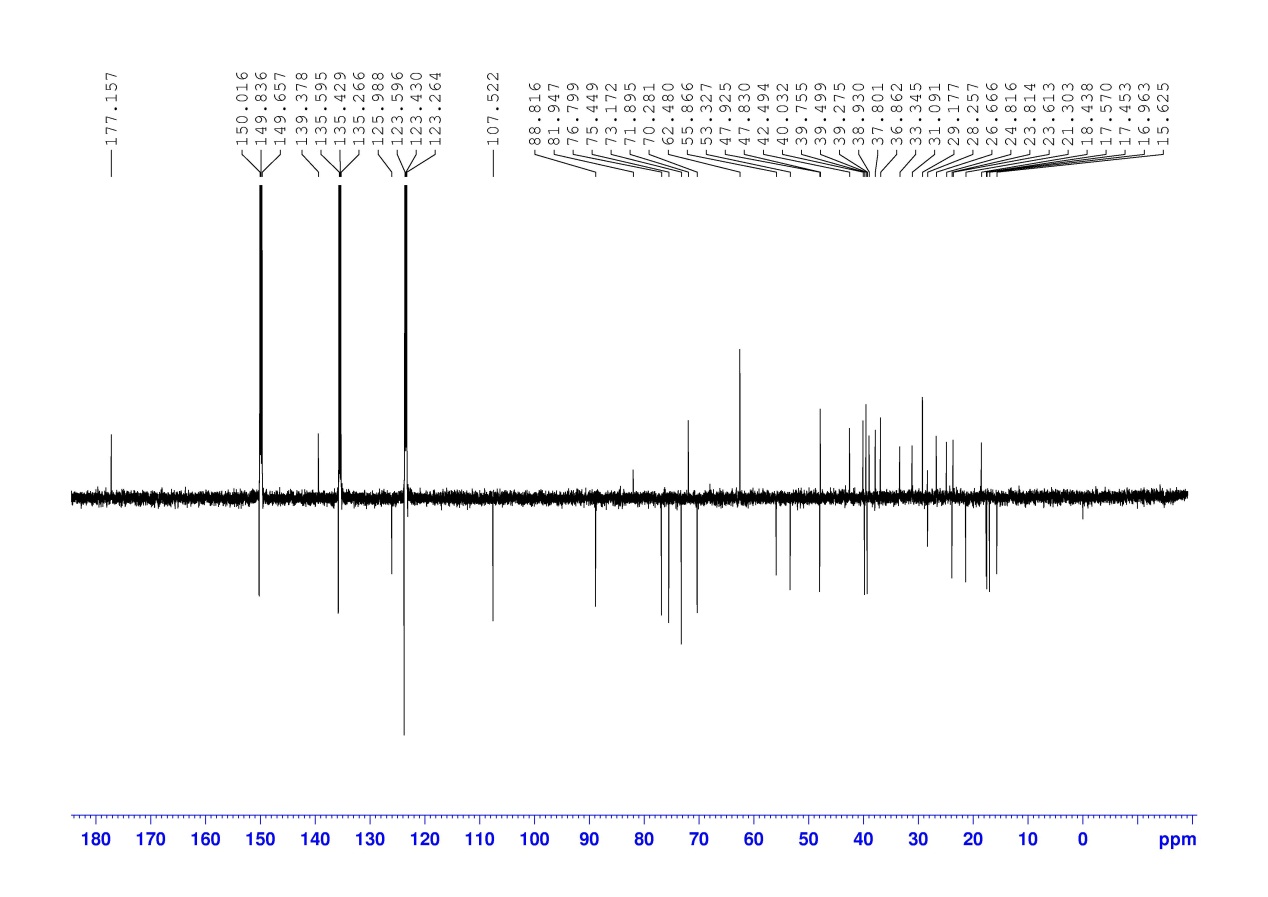


^13^C NMR of compound **V**


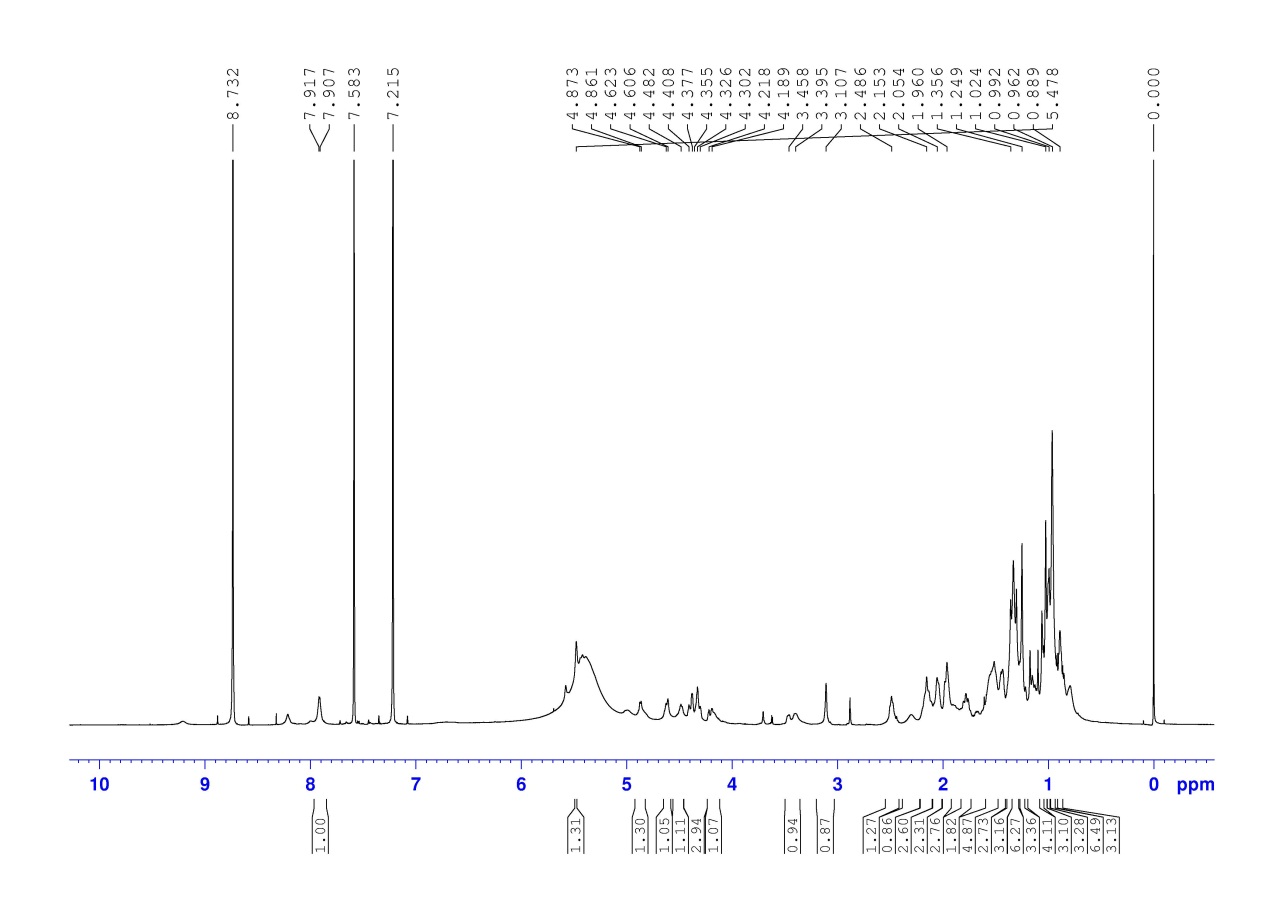


^1^H NMR of compound **VI** (**CE-P**)


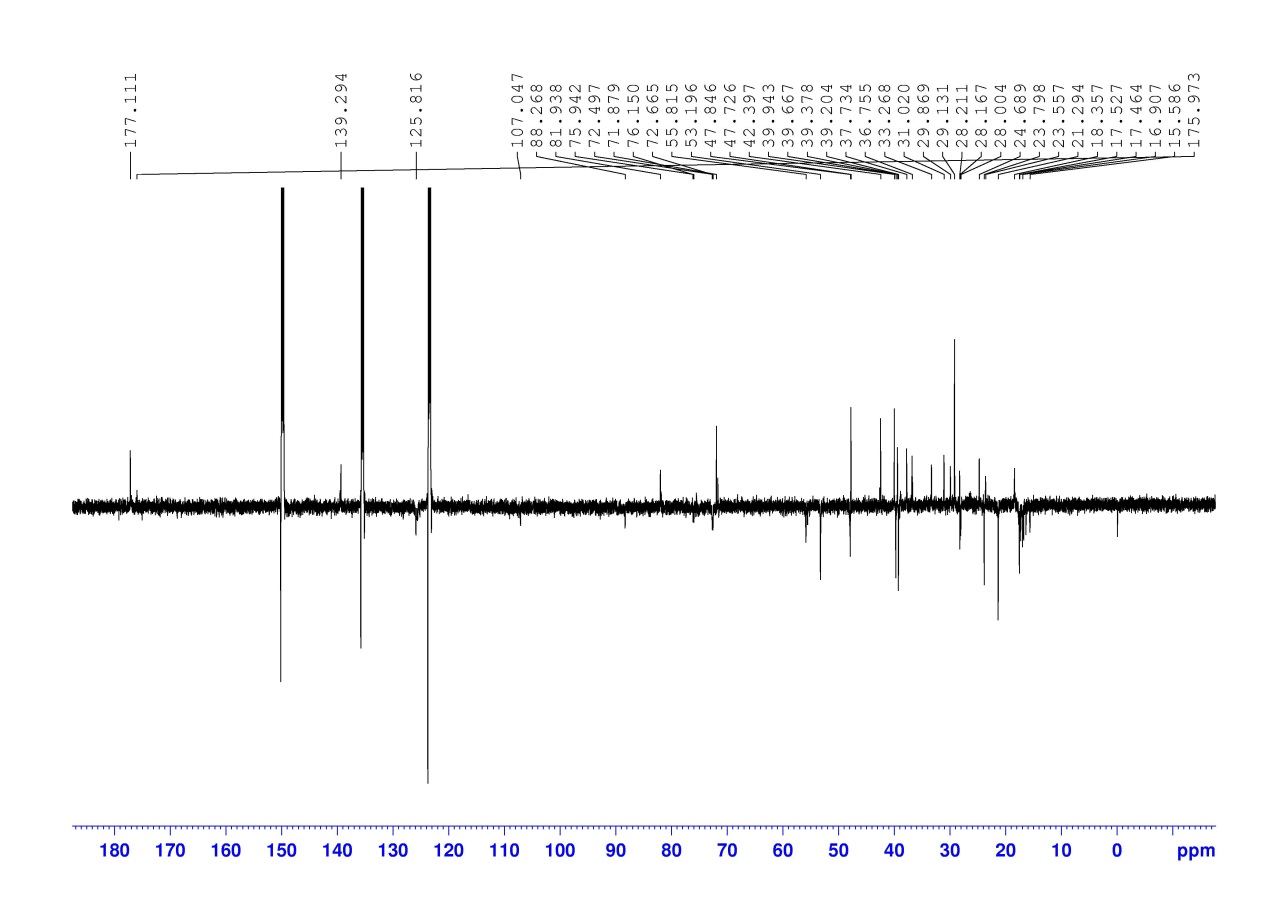


^13^C NMR of compound **VI** (**CE-P**)

ESI-MS of compound **VI** (**CE-P**)

**2,** **Protein Profiling of CE-P by click chemistry in HUVEC cell lysate.**

**Table S**1. Full lists of protein targets of **CE in HUVECs**

| NO | Accession | Description | Unique Peptides | MW [kDa] | emPAI | Score Mascot |
| --- | --- | --- | --- | --- | --- | --- |
| 1 | A8K9C4 | Elongation factor 1-alpha | 5 | 50.2 | 0.808 | 455 |
| 2 | Q562L9 | Actin-like protein | 1 | 11.5 | 9 | 329 |
| 3 | O75390 | Citrate synthase, mitochondrial | 4 | 51.7 | 0.403 | 301 |
| 4 | G8JLB6 | Heterogeneous nuclear ribonucleoprotein H | 3 | 51.2 | 0.25 | 280 |
| 5 | P22626 | Heterogeneous nuclear ribonucleoproteins A2/B1 | 3 | 37.4 | 0.425 | 255 |
| 6 | P08238 | Heat shock protein HSP 90-beta | 4 | 83.2 | 0.314 | 217 |
| 7 | V9HWB4 | Epididymis secretory sperm binding protein Li 89n | 1 | 72.3 | 0.134 | 213 |
| 8 | B2R4R0 | Histone H4 | 3 | 11.4 | 0.995 | 205 |
| 9 | P38159 | RNA-binding motif protein, Xchromosome | 3 | 42.3 | 0.162 | 200 |
| 10 | A0A0S2Z410 | Hydroxysteroid dehydrogenase 10 isoform 1 (Fragment) | 2 | 26.9 | 0.501 | 199 |
| 11 | P07737 | Profilin-1 | 2 | 15 | 0.585 | 197 |
| 12 | A8K4W0 | 40S ribosomal protein S3a | 1 | 29.9 | 0.186 | 194 |
| 13 | A0A024RAZ7 | Heterogeneous nuclear ribonucleoprotein A1, isoform CRA_b | 1 | 38.7 | 0.089 | 188 |
| 14 | F5H2F4 | C-1-tetrahydrofolate synthase, cytoplasmic | 2 | 110.5 | 0.095 | 181 |
| 15 | V9HWB8 | Pyruvate kinase | 3 | 57.9 | 0.212 | 171 |
| 16 | Q14103 | Heterogeneous nuclear ribonucleoprotein D0 | 2 | 38.4 | 0.202 | 168 |
| 17 | P13010 | X-ray repair cross-complementing protein 5 | 1 | 82.7 | 0.04 | 154 |
| 18 | F4ZW62 | NF45 | 5 | 43 | 1.015 | 145 |
| 19 | J3KPS3 | Fructose-bisphosphate aldolase | 3 | 39.8 | 0.407 | 142 |
| 20 | A0A140VJY2 | Testicular tissue protein Li 209 | 1 | 80.1 | 0.072 | 136 |
| 21 | B3KX96 | cDNA FLJ45003 fis, clone BRAWH3011623, highly similar to Heterogeneous nuclear ribonucleoproteins C | 2 | 32.3 | 0.16 | 133 |
| 22 | A0A087X0X3 | Heterogeneous nuclear ribonucleoprotein M | 1 | 77.5 | 0.035 | 132 |
| 23 | P07954 | Fumarate hydratase, mitochondrial | 4 | 54.6 | 0.266 | 131 |
| 24 | P19338 | Nucleolin | 2 | 76.6 | 0.076 | 131 |
| 25 | Q6UX06 | Olfactomedin-4 | 1 | 57.2 | 0.141 | 131 |
| 26 | V9HW22 | Epididymis luminal protein 33 | 1 | 70.9 | 0.139 | 126 |
| 27 | Q5EC54 | Heterogeneous nuclear ribonucleoprotein K transcript variant | 3 | 51 | 0.225 | 126 |
| 28 | P02753 | Retinol-binding protein 4 | 1 | 23 | 0.212 | 124 |
| 29 | A0A023T787 | RNA-binding protein 8A | 1 | 19.9 | 0.233 | 122 |
| 30 | P04181 | Ornithine aminotransferase, mitochondrial | 1 | 48.5 | 0.068 | 117 |
| 31 | P13639 | Elongation factor 2 | 2 | 95.3 | 0.102 | 113 |
| 32 | A8K401 | Prohibitin, isoform CRA_a | 2 | 29.8 | 0.212 | 111 |
| 33 | Q8IWZ3 | Ankyrin repeat and KH domain-containing protein 1 | 1 | 269.3 | 0.017 | 111 |
| 34 | Q59EJ3 | Heat shock 70kDa protein 1A variant (Fragment) | 1 | 77.4 | 0.089 | 110 |
| 35 | Q99536 | Synaptic vesicle membrane protein VAT-1 homolog | 2 | 41.9 | 0.202 | 108 |
| 36 | E9KL44 | Epididymis tissue sperm binding protein Li 14m | 1 | 82.9 | 0.037 | 103 |
| 37 | P49773 | Histidinetriad nucleotide-binding protein 1 | 1 | 13.8 | 0.668 | 101 |
